# A New Immortalized Human Alveolar Epithelial Cell Model to Study Lung Injury and Toxicity on a Breathing Lung-On-Chip System

**DOI:** 10.3389/ftox.2022.840606

**Published:** 2022-06-17

**Authors:** Arunima Sengupta, Nuria Roldan, Mirjam Kiener, Laurène Froment, Giulia Raggi, Theo Imler, Lea de Maddalena, Aude Rapet, Tobias May, Patrick Carius, Nicole Schneider-Daum, Claus-Michael Lehr, Marianna Kruithof-de Julio, Thomas Geiser, Thomas Michael Marti, Janick D. Stucki, Nina Hobi, Olivier T. Guenat

**Affiliations:** ^1^ Organs-on-Chip Technologies, ARTORG Center for Biomedical Engineering, University of Bern, Bern, Switzerland; ^2^ Alveolix AG, Swiss Organs-on-Chip Innovation, Bern, Switzerland; ^3^ Department of Pulmonary Medicine, Inselspital, Bern University Hospital, Bern, Switzerland; ^4^ Department for BioMedical Research DBMR, Urology Research Laboratory, University of Bern, Bern, Switzerland; ^5^ InSCREENeX GmbH, Braunschweig, Germany; ^6^ Department of Drug Delivery (DDEL), Helmholtz-Institute for Pharmaceutical Research Saarland (HIPS), Helmholtz Centre for Infection Research (HZI), Saarbrücken, Germany; ^7^ Department of Pharmacy, Biopharmaceutics and Pharmaceutical Technology, Saarland University, Saarbrücken, Germany; ^8^ Department of General Thoracic Surgery, Inselspital, Bern University Hospital, Bern, Switzerland

**Keywords:** alveolar epithelial cells, distal lung, lung-on-a-chip, SARS-CoV-2, cyclic stretch, lung inflammation, lung toxicity, AT1 and AT2

## Abstract

The evaluation of inhalation toxicity, drug safety and efficacy assessment, as well as the investigation of complex disease pathomechanisms, are increasingly relying on *in vitro* lung models. This is due to the progressive shift towards human-based systems for more predictive and translational research. While several cellular models are currently available for the upper airways, modelling the distal alveolar region poses several constraints that make the standardization of reliable alveolar *in vitro* models relatively difficult. In this work, we present a new and reproducible alveolar *in vitro* model, that combines a human derived immortalized alveolar epithelial cell line (^AX^iAEC) and organ-on-chip technology mimicking the lung alveolar biophysical environment (^AX^lung-on-chip). The latter mimics key features of the *in vivo* alveolar milieu: breathing-like 3D cyclic stretch (10% linear strain, 0.2 Hz frequency) and an ultrathin, porous and elastic membrane. ^AX^iAECs cultured on-chip were characterized for their alveolar epithelial cell markers by gene and protein expression. Cell barrier properties were examined by TER (Transbarrier Electrical Resistance) measurement and tight junction formation. To establish a physiological model for the distal lung, ^AX^iAECs were cultured for long-term at air-liquid interface (ALI) on-chip. To this end, different stages of alveolar damage including inflammation (via exposure to bacterial lipopolysaccharide) and the response to a profibrotic mediator (via exposure to Transforming growth factor β1) were analyzed. In addition, the expression of relevant host cell factors involved in SARS-CoV-2 infection was investigated to evaluate its potential application for COVID-19 studies. This study shows that ^AX^iAECs cultured on the ^AX^lung-on-chip exhibit an enhanced in vivo-like alveolar character which is reflected into: 1) Alveolar type 1 (AT1) and 2 (AT2) cell specific phenotypes, 2) tight barrier formation (with TER above 1,000 Ω cm^2^) and 3) reproducible long-term preservation of alveolar characteristics in nearly physiological conditions (co-culture, breathing, ALI). To the best of our knowledge, this is the first time that a primary derived alveolar epithelial cell line on-chip representing both AT1 and AT2 characteristics is reported. This distal lung model thereby represents a valuable *in vitro* tool to study inhalation toxicity, test safety and efficacy of drug compounds and characterization of xenobiotics.

## Introduction

The impairment of functional gas exchange at the alveolar epithelial barrier is a crucial determinant of the clinical outcome of various acute and chronic respiratory diseases, such as acute respiratory distress syndrome (ARDS) (Sapoznikov et al., 2019), emphysema in chronic obstructive pulmonary disease (COPD) (Moazed et al., 2016) and pulmonary fibrosis (PF) (Kulkarni et al., 2016). Such respiratory conditions are often detected at an advanced stage, when they are more difficult to treat. Hence, there is an urgent need for new investigative strategies to generate more effective therapeutics.

The alveolar microenvironment in the distal lung is a complex and dynamic structure that allows efficient gas exchange, determines immune responses to various environmental stimuli and forms a tight barrier that prevents any fluid accumulation into the lung airspace (Knudsen and Ochs, 2018). The alveolar epithelial lining forms the initial barrier against inhaled pathogens or xenobiotics. In a healthy lung, paracellular and transcellular barriers are formed by alveolar junctions that control the permeability to inhaled particles, toxins and pathogens. Thus, the destruction of the tight alveolar barrier by environmental stressors disrupts the delicate equilibrium of epithelial cells and alters their function and regulation of various signalling pathways (Olivera et al., 2007; Short et al., 2016). This highlights the significance of a concerted interplay between epithelial cell-cell communication and the stability of tight junction components in the alveoli. The alveolar epithelium comprises two distinct cell types: alveolar type I (AT1) and type 2 (AT2) pneumocytes which are tightly connected by intercellular junctions as well as other cell-to-cell connections. Physiologically mimicking parameters, such as culture at ALI, have been shown to significantly improve the alveolar character of lung alveolar primary cells and cell lines (A549) (Ravasio et al., 2011; Wu et al., 2018). On the other hand, breathing exposes the alveoli to continuous and differential levels of mechanical forces since early development (Waters et al., 2012). Experimental evidence has shown that *in vivo* like breathing motion (8–12% linear strain) plays an essential role to regulate alveolar cell differentiation (Liu et al., 2016; Li et al., 2018) and has the potential to induce regenerative responses in wound injury (Desai et al., 2008). In contrast, aberrant mechanical stress is a critical factor in the development of various lung diseases including acute lung injury, ARDS, lung fibrosis (Sehlmeyer et al., 2020) as well as ventilation induced mechanical trauma (Oeckler and Hubmayr, 2007).

Due to the lack of robust *in vitro* alternatives to study distal lung, and the inherent translational limitations of animal models, “new approach methodologies (NAMs)” are becoming increasingly relevant (Russell, 2004). Advanced *in vitro* models recapitulating important aspects of the alveolar niche promise to bridge the gap and provide a reliable alternative for drug safety as well as efficacy studies, toxicity profiling and precision medicine (Low et al., 2020). The organ-on-chip technology replicates key points of the dynamic alveolar microenvironment such as cyclic mechanical strain, perfusion and inflammatory or thrombotic pathomechanisms (Huh et al., 2010; Stucki et al., 2015; Jain et al., 2017; Stucki et al., 2018; Felder et al., 2019; Thacker et al., 2020; Huang et al., 2021; Si et al., 2021). Besides, the recent inclusion of 3D breathing motion (10% linear strain), ALI culture conditions, biological scaffolds and use of primary human alveolar epithelial cells (AECs), has brought the lung-on-chip technology closer to physiological dimensions (Stucki et al., 2015; Stucki et al., 2018; Zamprogno et al., 2021).

Notwithstanding the complexity of organs-on-chip, a critical challenge remains the procurement of a suitable cell source. Primary human AECs continue to be one of the most reliable alveolar cell models reflecting the *in vivo* situation in terms of alveolar phenotype and barrier formation (Elbert et al., 1999), molecule absorption and ion-transport studies (Bove et al., 2010; Bove et al., 2014). Their combination with lung-on-chip models or their use as alveolar organoids has predictive and translational applications, which have been accelerated and successfully put to test in drug screening and pathway studies, particularly due to the COVID-19 pandemic (Zhang et al., 2020; Thacker et al., 2021; Domizio et al., 2022) (Huang et al., 2020; Katsura et al., 2020; Youk et al., 2020). On the other hand, iPSC-derived AECs exhibit highly relevant alveolar features and are fitting on-chip candidates (Katsumiti et al., 2020; Van Riet et al., 2020). However, they present a labour-intensive generation and require highly experienced personnel. Primary AECs have their limitations too, including their restricted access, inability to be expanded and donor-specific response. Thus, alternative models capturing relevant features of these cells, combined with elements of the dynamic alveolar microenvironment, offer an opportunity for studies requiring a stable alveolar phenotype at instances where primary cell material proves limiting.

Alveolar epithelial cell lines have been used since the late 80’s. A549 and NCI-H441 are still the most widely used human AEC cell lines in pharmaceutical research for lung cancer, asthma, COPD and fibrosis despite their carcinogenic origin (Giard et al., 1973; Brower et al., 1987; O’Reilly et al., 1988). Conversely, viral transduced primary AT2 cells have led to the generation of AT1-like cell lines, including TT1cells (Kemp et al., 2008; van den Bogaard et al., 2009; Katsumiti et al., 2020), or the recently established hAELVi cells (Kuehn et al., 2016). These cell lines display different features of ATI cells phenotype, such as a flattened and extended morphology (Kemp et al., 2008), or TER-forming barrier properties, respectively (Kuehn et al., 2016; Metz et al., 2020). Besides, both have been successfully implemented in the study of lung inflammation, showing cytokine responses upon proinflammatory triggers such as lipopolysaccharide (LPS), interferon gamma (IFN-γ) or tumor necrosis factor alpha (TNF-α) (van den Bogaard et al., 2009; Metz et al., 2020). Nevertheless, none of these models fully comprised functionally tight barrier, robust long-term alveolar character with AT1 and AT2-like cells, ultra-thin basement membrane and breathing dynamics.

To address this need, we report in this work an *in vitro* system including an immortalized human alveolar epithelial cell line (^AX^iAEC) combined with the ^AX^lung-on-chip system (AlveoliX, Switzerland). Primary cell-derived ^AX^iAECs were characterized by FACS for typical epithelial and AT1/AT2 markers. ^AX^iAECs cultured on-chip were further inspected for their alveolar cell-specific gene expression profile as well as the presence of cellular host factors required for SARS-CoV-2 (severe acute respiratory syndrome Coronavirus 2) infection. Subsequently, the impact of physiological levels of cyclic strain and ALI culture conditions on cell phenotype was examined by gene expression and protein profiling. Finally, ^AX^iAECs cultured on-chip were exposed to a well-characterized pro-fibrotic growth factor, transforming growth factor β1 (TGFβ1), and a pro-inflammatory stimulus, bacterial LPS, to evaluate the responsiveness and suitability of the model to study different stages of lung injury and its potential application for inhalation toxicology studies and drug safety and efficacy testing.

## Methods

### Design and Handling of the ^AX^Lung-On-Chip System

**FIGURE 1:**
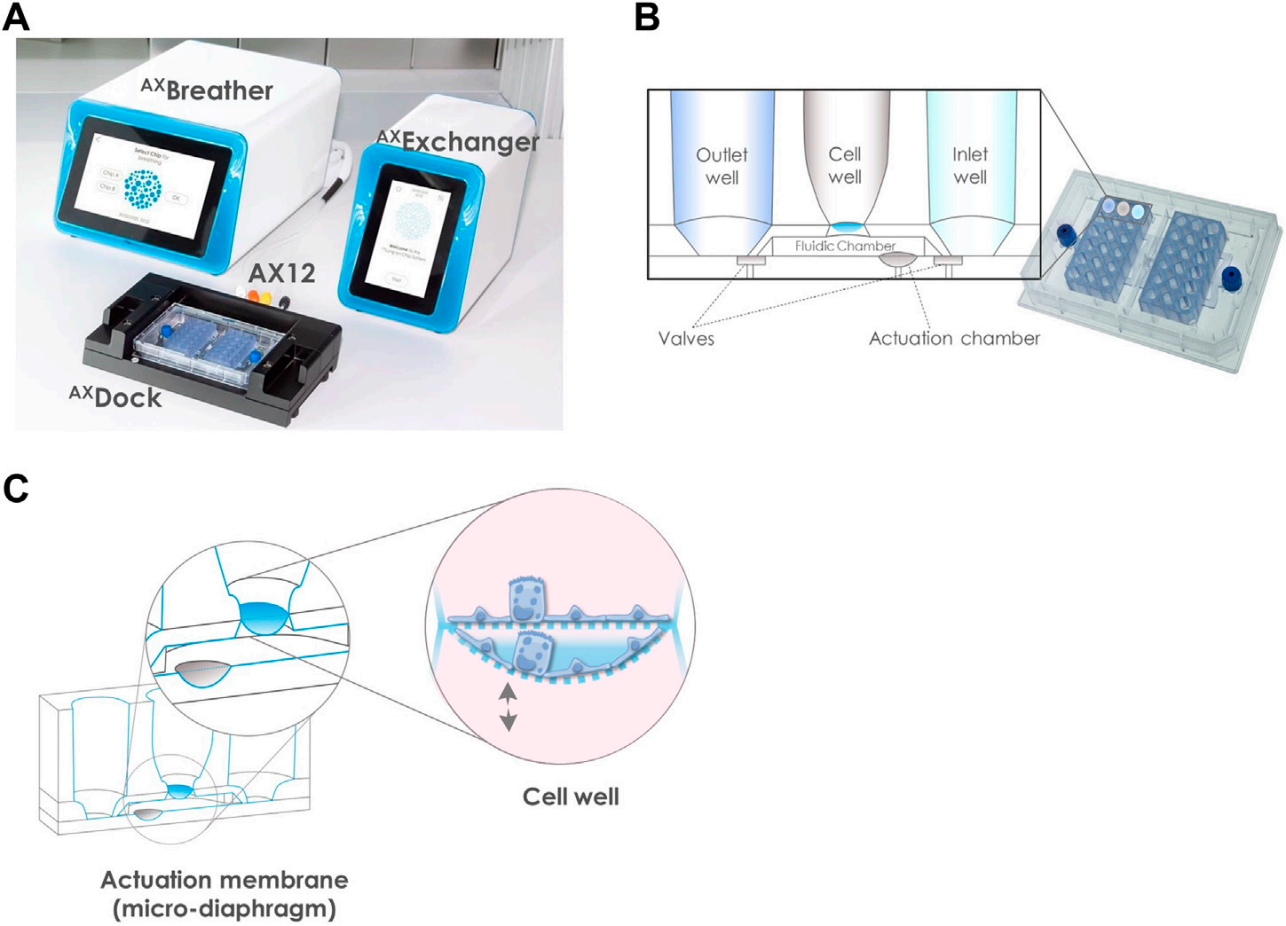
Overview of the ^AX^Lung-on-chip System. **(A)** including the Lung-on-chip (AX12), two electro-pneumatic modules: ^AX^Exchanger and ^AX^Breather, and the interface platform: ^AX^Dock. **(B)** Detail of the AX12 comprised of two chips. Based on a SBS footprint (standard 96-well plate layout 127 mm × 85 mm), the wells are positioned at equal distance of 9 mm. The chip cross-section schematic shows that one unit comprises of an inlet and an outlet well, connected through microfluidic channels and pneumatic valves to the cell compartment with the 3.5 µm ultrathin membrane of porous density 8 × 10^5^ pores/cm^2^ (blue). The two valves define the medium flow via the control of the ^AX^Exchanger, for which 200 µl of medium are pipetted in the inlet wells. **(C)** Principle of the breathing function. In the fluidic chamber of a volume ∼80 μl, the actuation membrane (grey), acting as a micro-diaphragm, is cyclically stretched in 3D by the ^AX^Breather. This movement is transferred to the cells cultured on the cell well membrane (blue). The cell barrier recreated on the ultrathin porous membrane is biomechanically stimulated with a physiological 3D-stretch of 10% linear strain and 0.2 Hz frequency.

The ^AX^Lung-on-chip system (AlveoliX, Switzerland) consists of the Lung-on-Chip, called AX12, two electro-pneumatic devices and two interface units (Figure 1A). The electro-pneumatic modules, the ^AX^Exchanger and ^AX^Breather, are connected to the AX12 via the interface platform, the ^AX^Dock, respectively, in the cell culture hood and in the incubator. The AX12 placed on the ^AX^Dock is controlled by the touchscreen interface of the ^AX^Exchanger and ^AX^Breather. The AX12 has a SBS footprint (96-well plate format) and comprises of two modular chips, supported by a plate, that contains six individual units each. Each unit is composed of an inlet, a cell compartment and an outlet that are connected on the basal side by microfluidic channels and pneumatically controlled valves. The cell compartment comprises an ultrathin (3.5 µm) and elastic porous membrane (3 μm, 8 × 10^5^ pores/cm^2^) made of biocompatible silicone. This membrane separates the cavity into an apical cell well and a basolateral fluidic chamber (Figure 1B). The two-part design (modular chips and plate) of the AX12 enables the seeding of the cells directly on either side of the ultrathin membrane.

The chip closing and filling of the basolateral compartment are next performed. Briefly, a drop of cell culture media (with or without cells) is pipetted on the basolateral side of the membrane. Then, each chip is flipped and screwed onto the AX12 plate (blue screw, Figure 1B). The AX12 is then inserted into the ^AX^Dock, and inlet wells are filled up with cell culture media. The chip initial filling is triggered by pressing the “Initial filling” action on the ^AX^Exchanger: negative and positive pressure is generated to open and close the valves inducing a pumping action, which allows the medium of the inlet to flow into the basal chamber, and subsequently to the outlet well. After basolateral filling, cells are seeded on the apical side of the membrane.

For medium exchange, the AX12 is positioned in the ^AX^Dock. The inlet, outlet, and cell well are emptied and fresh cell culture media is pipetted into the inlet well. Similarly, as for the initial filling, medium exchange is performed via the “Medium Exchange” function of ^AX^Exchanger: negative pressure is generated to open the valves and the medium is exchanged by hydrostatic and surface tension forces. The nutrient-exhausted medium on the apical side is replaced with fresh medium by direct pipetting.

To initiate the breathing function, the AX12 is positioned within the ^AX^Dock within the incubator. 3D cyclic stretch (10% linear strain, 0.2 Hz) is initiated by touchscreen control on the ^AX^Breather. This automatically leads to the closing of the fluidic chamber and the deflection of the microdiaphragm by the generation of cyclic negative pressure, which recreates the *in vivo* breathing motions (Figure 1C). Static (non-breathing) and dynamic (breathing) conditions can be set simultaneously on one AX12, as the two chips are controlled independently.

### Cells and Cell Culture

The alveolar epithelial cell line (^AX^iAECs) was derived from primary human AECs isolated from resected lung tissue, immortalized with InscreeneX’ CI-Screen technology (Lipps et al., 2018), and provided by AlveoliX (Switzerland). ^AX^iAECs were cultured and expanded in AX Alveolar Epithelial Medium (AlveoliX, Switzerland) and used between passages 23 and 30. AX12 were provided precoated with ECM (AlveoliX, Switzerland). On-chip, the cells were maintained in AX Alveolar Barrier Medium (AlveoliX, Switzerland), supplemented with 1% penicillin-streptomycin (ThermoFischer Scientific, Switzerland).

For the co-culture experiments (AX co-culture Biomodel), ^AX^iAECs were seeded apically, and primary human lung microvascular endothelial cells (hLMVEC) at the basolateral side of the membrane. AX E2 Alveolar Barrier Medium (AlveoliX, Switzerland) was used for both cell types. Peripheral blood mononuclear cells (PBMCs) were introduced at the initiation of the inflammation experiments. All cell manipulations were performed under sterile laminar flow conditions and cells were maintained at 37°C, 5% CO_2_. Cell culture medium was replaced every 2–3 days. For monoculture studies on-chip, ^AX^iAECs cells were seeded at a density of 4 × 10^5^ cells per cm^2^ on the apical side of the membrane. The cells were incubated for 24 h allowing them to adhere to the membrane and reached confluence after 48 h. For the co-culture with endothelial cells, hLMVEC were first expanded in AX endothelial medium. Then, harvested hLMVEC were seeded on the basolateral side of each membrane followed by 2 h incubation to promote cell attachment. Chips were consecutively closed and filled with AX E2 Alveolar Barrier Medium using the initial filling function on the ^AX^Exchanger. ^AX^iAECs were subsequently seeded on the apical side of the membrane at a density of 4 × 10^5^ cells per cm^2^ in medium. ^AX^iAECs cell seeding is considered as day 0 in the study (D0).

### TER Measurement

To assess barrier formation, transbarrier electrical resistance (TER) measurements were started 48 h after cell seeding. TER measurements were taken every 2 days using a commercially available 96-well plate electrode (STX100MC96; World Precision Instruments) and an Epithelial Volt/Ohm Meter (EVOM3; World Precision Instruments). TER was measured in mono and co-culture conditions for up to 30 days. The unique semi-open design of the AX12 allows the STX100MC96 electrode to tightly fit in between the outlet well and the cell well which enables precise and consistent positioning of the electrodes and avoid handling variations. The microfluidic channel design: widths between 400 and 600 µm, height of up to 1,000 µm and length of no more than 1,000 µm, enables a low chip induced TER background essential for reliable on-chip measurements.

Before measurement, the electrodes were sterilized with 70% v/v ethanol, rinsed and equilibrated in DPBS (Thermo Fischer Scientific, Switzerland) at room temperature. To measure TER, the AX12 was placed on the ^AX^Dock in the cell culture hood. The electrodes were properly positioned and the “TER measurement” function was initiated on the ^AX^Exchanger, enabling valve opening on the microfluidic plate. For cells in ALI, pre-warmed cell culture medium was added 15 min before measuring TER. The background TER (Ω) was measured on a porous membrane with no cells. Background subtracted TER (Ω) values were then multiplied by the surface area of the cell culture well (0.071 cm^2^ on-chip) to obtain the final TER reading in Ω cm^2^.

### Cell Culture Treatments

^AX^iAECs were stimulated with Transforming Growth Factor β1 (TGFβ1) upon barrier formation. Lyophilized recombinant human TGFβ1 Protein (C# 240-B, R&D) was reconstituted in sterile 4 mM HCl containing 0.1% BSA following the manufacturer’s protocol. Cells were stimulated apically at T0 time-point (day 25 on-chip) with a final concentration of 5 or 10 ng/ml diluted in the cell culture medium (AX Alveolar Epithelial medium) for 72 h (T3). Control (vector treated) cells were added with reconstitution medium without TGFβ1. Following treatment, cells were harvested 3 days after TGFβ1 stimulation (T3).

^AX^iAECs/hLMVEC (AX co-culture Biomodel) were stimulated with 0.1 μg/ml LPS from E-Coli 026:B26 (Sigma Aldrich, Germany) in the presence of peripheral blood mononuclear cells (PBMCs). PBMCs were administered in the basolateral compartment via medium exchange. PBMC and LPS instillation was performed at L0 time-point (day 25 on-chip) and maintained for 3 days. TER was monitored every 24 h and ELISA of the apical supernatants were assessed after 72 h of treatment (L3).

### Flow Cytometry Analysis

Flow cytometry was performed on cells prior culture on-chip, denoted as D0 in this study. A549 and ^AX^iAECs were expanded in ECM coated T75 flasks (Greiner Bio-One, Switzerland). Expanded cells were harvested before staining and resuspended in FACS staining buffer. Freshly isolated AT2 cells were used as a positive control for alveolar markers. Cells were incubated with the following fluorescently conjugated human monoclonal antibodies from Miltenyi Biotec: CD326 (Epcam)-APC (130-111-117), CD271 (LNGFR)-PE-Vio770 (130-112-792), MUC1- PE-Vio770 (130-106-838). HTI-56 and HTII-280 from Terrace Biotech were added to the original panel and coupled, respectively, with Goat anti-Mouse IgG-PE (C#12-4010-82, eBioscience, Switzerland) and Goat anti-Mouse IgM-PE (C# M31504, Invitrogen, Switzerland) secondary antibodies in a sequential incubation. Cells were incubated on ice in the dark for 30 min. To exclude dead cells and debris, Zombie Green staining was performed prior to immunostaining. Cell acquisition was achieved using a BD FACS SORP LSRII. For analysis, a minimum of 1 × 10^4^ events were collected and analyzed using FlowJo software version 10.8.

**FIGURE 2:**
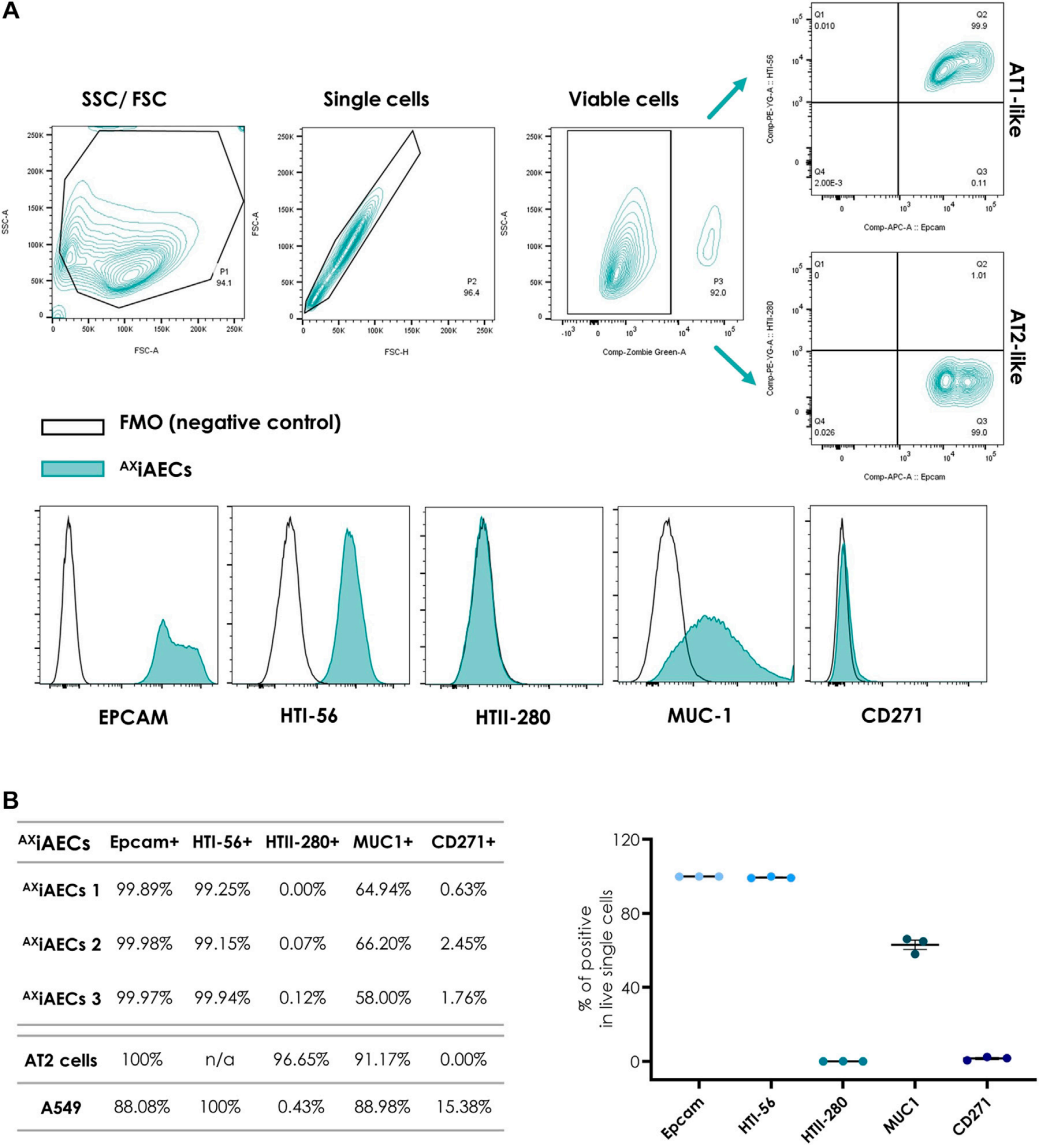
Cell surface phenotype analysis of ^AX^iAECs by flow cytometry at D0 **(A)** FACS gating strategy for identifying distinct alveolar epithelial cell surface markers as indicated in *Methods*. Fluorescence minus one (FMO) controls were used for all quantifications. Data are representative of three independent experiments. **(B)** Expression of cell surface markers is shown from three independent experiments with ^AX^iAECs and one experiment with human AT2 cells and A549 cells. Results are expressed as a percentage of the live single-cell population. Data are represented as mean ± SEM.

Fluorescence minus one (FMO) controls were used for gating strategy. Forward scatter (FCS) and side scatter (SSC) profiles allowed the exclusion of debris and doublets (P1 and P2, Figure 2A). Dead cells were excluded based on the Zombie Green gate. Analysis was performed on live single cell population (P3, Figure 2A).

### qRT-PCR

Total RNA was isolated and purified using the Direct-zol™ RNA Microprep kit following manufacturer’s instructions (Zymo Research, Switzerland). Cells were lysed with the supplied TRI Reagent. RNA concentration and purity were analyzed with a Nano-Drop Spectrophotometer (ThermoFischer Scientific, Switzerland). cDNA preparation was performed using the Super Script III Reverse Transcriptase kit (Life Technologies, Switzerland) according to manufacturer’s instructions. qRT-PCR reactions were performed in triplicates with SYBR^®^ Select Master Mix (Thermo Scientific) in an ABI7500 Fast (Applied Biosystems) real-time PCR system. Target gene expression was normalized to housekeeping gene expression (HPRT). The primer sequences are provided in Supplementary Table S6.

### Immunofluorescence Staining and Imaging

Cells cultured on AX12 were fixed with 4% paraformaldehyde in DPBS. The modular chips were unscrewed from the plate to separate the fluidic and pneumatic parts. Chips were then opened with the ^AX^Disassembly tool (AlveoliX, Switzerland) to dissociate the chip bottom part with the embedded membrane prior to staining and mounting.

Following permeabilization with 0.1% Triton X-100 (Sigma-Aldrich, Germany), cells were blocked with blocking buffer solution (2% BSA in DPBS- (Sigma-Aldrich, Germany)). Mono-culture staining was performed with: mouse anti-ZO-1 antibody (C# 33-9100, Fisher T Scientific), rabbit anti-mature SP-C antibody (C# WRAB- 76694, Seven Hills Bioreagents), mouse anti-HTI-56 (C# TB-29AHTI-56, Terrace Biotech), rabbit anti-MUC1 (C#NBP1-60046, Novus Biologicals), Epcam conjugated antibody (C# 130-111-117, Milteny Biotec), rabbit anti-HOPX (C# ab106251, Abcam), rabbit anti-ABCA3 (C# ab99856, Abcam), mouse anti-PECAM1 (#3528 Cell Signaling). Human ACE-2 Alexa Fluor 647 conjugate (C# FAB9332R, R&D Systems) and mouse anti-TRMPSS2 (clone P5H9-A3; C# sc-10184, Santa Cruz). The primary antibodies were diluted in 2% BSA/PBS and incubated overnight at 4°C. Secondary antibodies were used as follows: donkey anti-mouse Alexa Fluor 488 (C# A21202, Invitrogen), donkey anti-rabbit Alexa Fluor 568 (C# A10042, Invitrogen), were diluted 1:2,000 in 2% BSA/PBS and incubated 2 h at RT. Nuclei were stained with DAPI (C#D1306, Invitrogen). The actin cytoskeleton of the cells was visualized using the conjugated Alexa Fluor 647 phalloidin stain (C# PHDN1-A, Cytoskeleton, Inc.). Lastly, the stained membranes were sealed between two glass coverslips using mounting medium (C# F6182, Sigma-Aldrich).

Images were obtained using a confocal laser scanning microscope (Zeiss LSM 710), or Nikon Eclipse Ti-E Spinning Disk using appropriate filter settings. For fluorescent intensity calculation, Zen Blue software v2.1 (Zeiss) was used to obtain background corrected mean fluorescence intensity (MFI) for each channel of interest. To obtain comparable results from different area of interests on the membrane, identical settings for the optical and digital gain, area of focus and laser intensity was maintained. Finally, the mean fluorescence intensity (MFI) of the test channel (green channel, ZO-1) was normalized with the MFI of the blue channel (stained with DAPI).

### ELISA

Collected supernatants were analyzed for IL-8 secretion using the 4-Plex ProcartaPlex custom (Thermo Scientific, Switzerland) for the Bio-Plex from Bio-Rad following manufacturer recommendations.

### Statistical Analysis

All data are presented as mean ± standard error of mean (SEM). For AX12 experiments, “N” represents the experimental repetitions and “*n*” represents the number of individual wells accounted for across all experiments. For gene expression analysis, “*n*” represents the pool of three to four wells per experimental repetition. Two-tailed unpaired Student’s *t*-test was used to assess significant differences using GraphPad Prism v8.0 software. Statistical significance was defined as follows: *p < 0.05, **p < 0.01, ***p < 0.001. The exact number of repeats performed for each experiment is indicated in the corresponding figure legends.

## Results

### Identification of Alveolar Epithelial Subsets by FACS

^AX^iAECs were investigated for AT1 and AT2 cell-specific protein expression by flow cytometry at D0. In parallel, human primary AT2 cells isolated from lung resections and the tumor derived, alveolar cell line A549, where analysed for comparison (Figure 2C). Epcam is an epithelial specific cell adhesion molecule (Litvinov et al., 1997), ^AX^iAECs and freshly isolated AT2 cells were 100% Epcam+, whereas A549 cells had mixed cell populations (Epcam+, 88.08%). Among the Epcam+ population, most ^AX^iAECs cells were HTI-56+ cells (∼99.44%), an AT1 specific cell marker, indicating a predominant AT1-like character (Dobbs et al., 1999). To investigate the AT2-like population, we screened the cells for the AT2 cell specific marker HTII-280. Our results revealed that it was predominant on primary freshly isolated AT2 cells (HTII-280+, ∼96.65%), but could not be detected on ^AX^iAECs (∼0.06%) nor on A549 cells (0.43%) (Gonzalez et al., 2010). We further investigated Mucin1, a marker expressed by AECs, known to have a higher presence in AT2 cells (Jarrard et al., 1998). Our findings were consistent with the data reported in the literature: 91.17% of the AT2 cells were positive for Muc1, whereas Muc1+ A549 cells represented 88.98% of the total population. More than 2/3 of the Epcam+ ^AX^iAECs were positive for Muc1 (Muc1; ∼63.04%). Finally, we investigated the presence of Nerve growth factor receptor (NGFR/CD271) as a negative control, given that it is a typical marker for upper airway cells. AT2 cells (NGFR, 0%) and ^AX^iAECs were negative for this marker (∼1.61%), whereas a fraction of A549 cells was positive (15.38%). Altogether, our results support the alveolar character of the ^AX^iAECs, with predominant AT1-like characteristics when compared to AT2 cells (Figure 2C). A549, however, consisted in a mixed population comprising alveolar epithelial, Epcam- and NGFR+ cells.

### Molecular (Alveolar) Characterization of the ^AX^iAECs On-Chip

**FIGURE 3:**
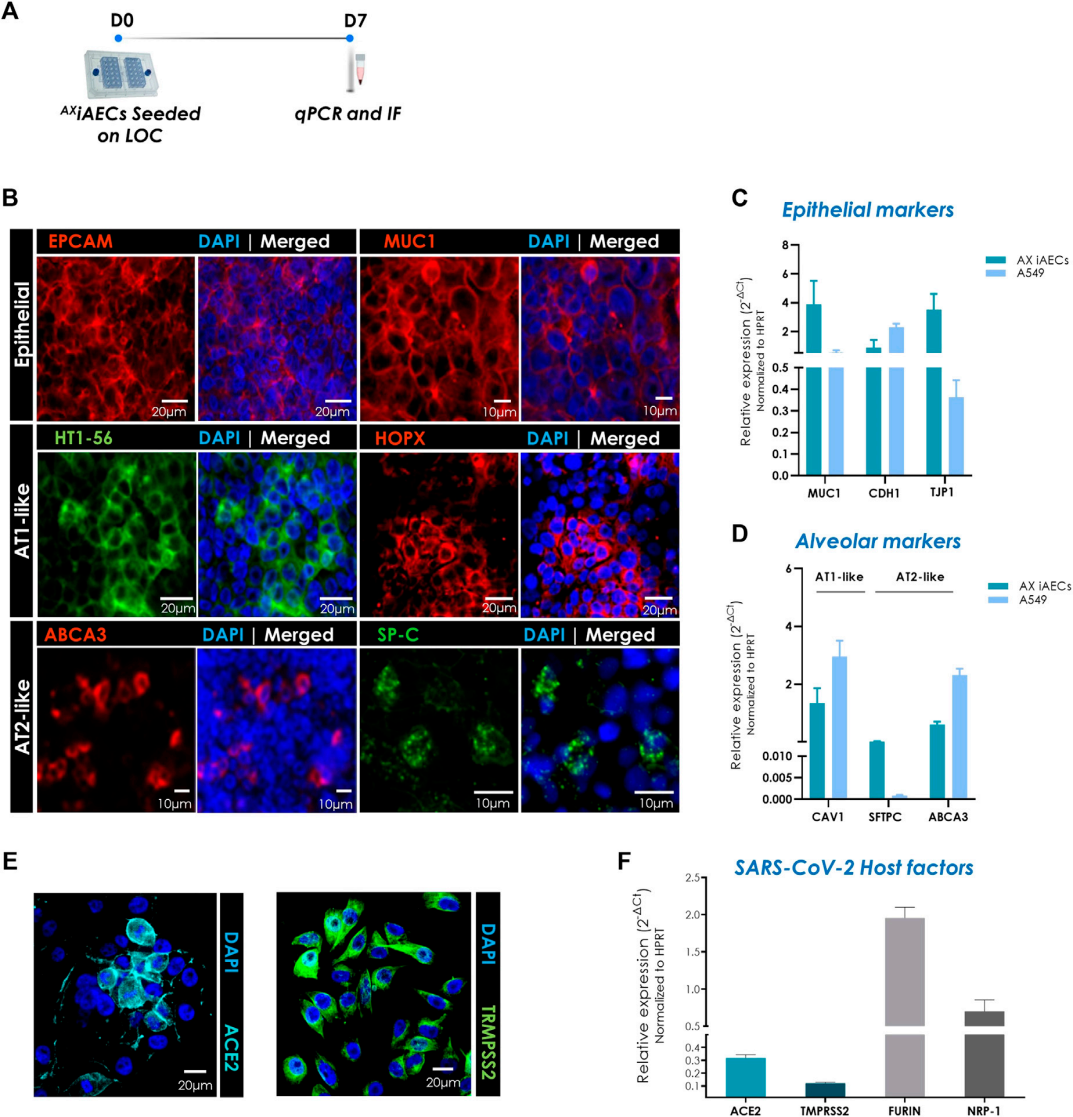
Expression of distinct alveolar epithelial cell markers in ^AX^iAECs cultured on ^AX^Lung-on-chip. **(A)** Timeline and schematic of the ^AX^iAECs cultured on the AX12. **(B)** Representative immunofluorescent staining for ^AX^iAECs fixed after 7 days cultured in AX12 (D7), probed for epithelial: EPCAM (red), MUC1 (red); AT1-like: HTI-56 (green), HOPX (red). and AT2-like markers: SP-C (green), ABCA3 (red). Nuclei were stained with DAPI (blue). Scale bar is provided with each image. **(C)** Relative gene expression of distinct epithelial and **(D)** alveolar genes in A549 cells (N = 1; *n* = 3) and ^AX^iAECs cultured on ^AX^Lung-on-chip (N = 2; *n* = 4) for 7 days. **(E)** ^AX^iAECs stained for ACE2 (cyan) and TMPRSS2 (green). **(F)** qPCR results show gene expression of SARS-CoV-2 host factors in ^AX^iAECs (N = 2; *n* = 4) after 7 days of culture in AX12. Data are shown as mean ± SEM, *p < 0.05, **p < 0.01, ***p < 0.001.

To investigate the cellular identity of the ^AX^iAECs cultured on-chip in terms of alveolar cell fate, alveolar and epithelial markers were studied at day 7 at the gene and protein level (Figure 3A).

Fluorescence imaging revealed that ^AX^iAECs expressed epithelial cell-specific proteins like Epcam and mucin 1 (MUC1) following 7 days of culture at the protein level. Furthermore, the AT1 cell-specific markers HOP Homeobox (HOPX) (Liebler et al., 2016) and HTI-56 (Dobbs et al., 1999) were also expressed, supporting an AT1-like character (Figure 3B). On the other hand, the canonical AT2 cell-specific surfactant protein C (SP-C) (Glasser et al., 1987; Warr et al., 1987) and the ATP-binding cassette sub-family A member 3 (ABCA3) (Mulugeta et al., 2002) were additionally observed on these cells, highlighting the presence and coexistence of both alveolar epithelial types on the AX12.

We further evaluated the alveolar character of the ^AX^iAECs-on-chip at the gene level by RT-qPCR (Figures 3C,D). As a reference, we used A549 as a conventional *in vitro* model of the distal airway often used to assess cytotoxicity and (pro)inflammatory responses (Öhlinger et al., 2019; Barosova et al., 2021). Our results confirmed that both cell types, ^AX^iAECs and A549, present similar transcript levels of the epithelial genes E-cadherin-1 (CDH1) after 7 days cultured on-chip. However, both the epithelial mucin MUC1 (Relative/Rel. expression ^AX^iAECs 3.87 vs. A549 0.54; *p*-value 0.0279 and the tight junction protein 1 (TJP1) (Rel. expression ^AX^iAECs 3.50 vs. A549 0.36; *p*-value 0.0402), demonstrated a higher expression on ^AX^iAECs than in A549. Genes associated to the alveolar character (Caveolin1; CAV1 and ABCA3) were in a similar range for both cell lines, with surfactant protein C (SFTPC) gene showing a higher expression on ^AX^iAECs. Besides, SFTPC gene expression increased after culture time on-chip in contrast to A549 cells (D7, Figure 3D vs. D0, Supplementary Figure S1D). Overall, the gene expression profile of ^AX^iAECs on-chip showed transcripts levels in range with whole lung tissue and primary AT2 cells for the studied markers, with the exception of the highly AT2-specific SFTPC. A549 cells exhibited a similar gene marker profile to ^AX^iAECs at D0 (Supplementary Figures S1C,D). However, marked differences were observed when comparing A549 cultured on inserts, a widely adopted *in vitro* model (Barosova et al., 2021), with their culture on the AX12 over 7 days (Supplementary Figures S1E,F). Culture on-chip led to a general improvement in the alveolar and epithelial character.

At the protein level, A549 cells showed expression of Epcam and HT1-56 with a few cells expressing ABCA3, in line with our qPCR observations (Supplementary Figure S2A). These cells, however, quickly led to the formation of thick multilayers (Supplementary Figure S2B), whereas ^AX^iAECs remained stable up to D14 on-chip (Supplementary Figure S2B).

The zoonotic transmission of the coronavirus has been a crucial concern in the ongoing SARS-CoV-2 pandemic. Recent studies have highlighted certain key host factors that are crucial for the entry and promotion of SARS-CoV-2 infection in human host cells including Angiotensin I Converting Enzyme 2 (ACE2) and Transmembrane Serine Protease 2 (TRMPSS2) (Baggen et al., 2021). To further characterize our model of the distal airway, we analyzed the expression of ACE2 and TRMPSS2 by immunofluorescence staining and gene expression at D7. Our results showed that ^AX^iAECs express both ACE2, TRMPSS2 at the protein and gene level (Figures 3E,F), and confirmed the gene expression of other genes relevant for SARS-CoV-2 infection such as Neuropilin 1 (NRP1) and Furin/Paired Basic Amino Acid Cleaving Enzyme (FURIN) on-chip over time (Figure 3F).

### Robust Long-Term Barrier Formation On-Chip

**FIGURE 4:**
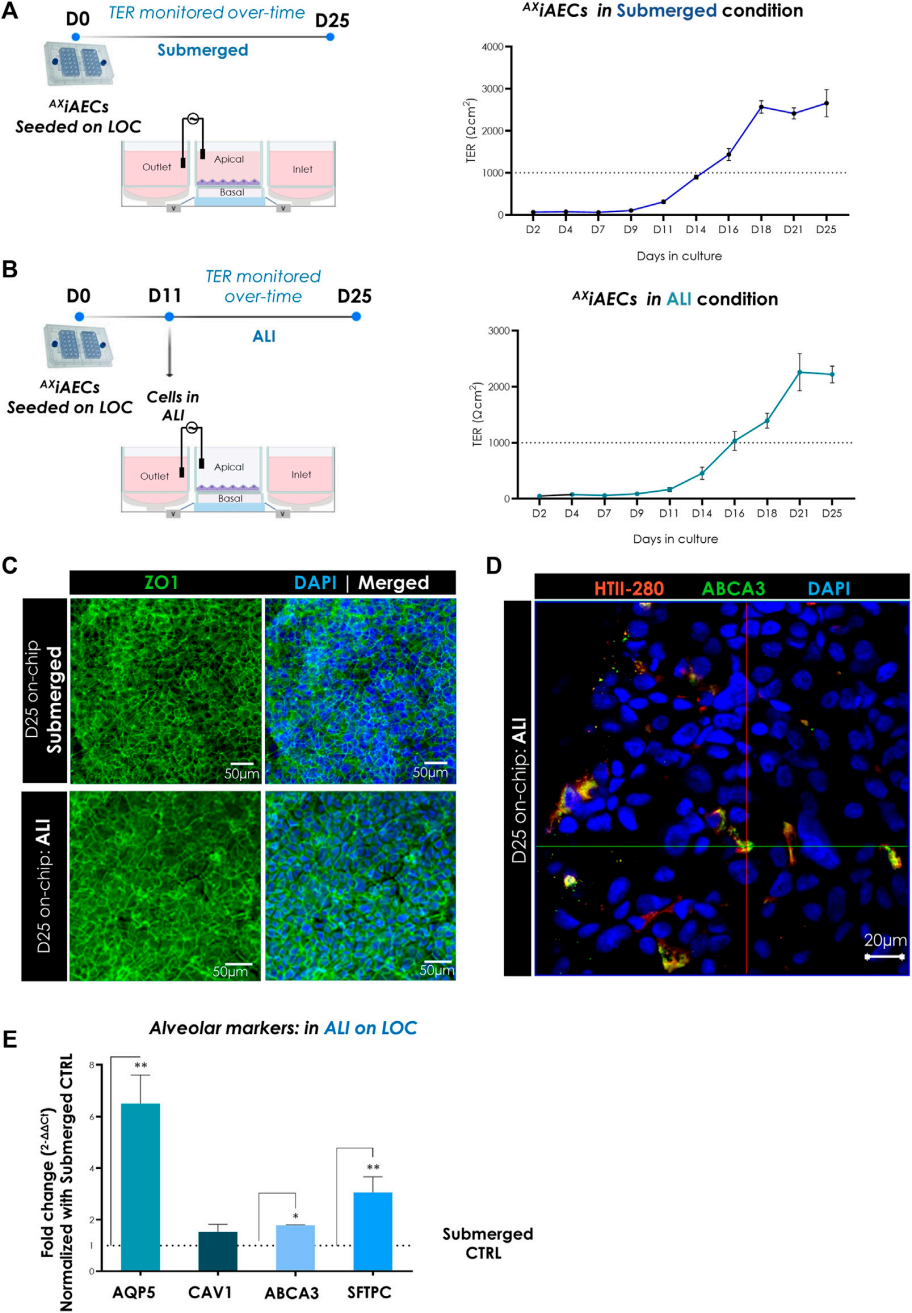
Characterization of barrier formation on-chip. **(A)** Timeline and overall schematic of the ^AX^iAECs cultured in submerged state on the AX12. TER (Ω cm^2^) values of ^AX^iAECs in submerged conditions on-chip until day 25 (D25) are shown. TER values above 1,000 Ω cm^2^ were recorded around day 16 (N = 3; *n* = 10). **(B)** Timeline and overall schematic of the ^AX^iAECs cultured in ALI condition starting from D5 onwards on-chip. TER (Ω cm^2^) values of ^AX^iAECs in ALI on-chip maintained until day 25 (D25) are shown. TER values above 1,000 Ω cm^2^ were recorded around day 16 (N = 3; *n* = 9). **(C)** Representative image for ^AX^iAECs in submerged and ALI conditions, fixed after 25 days culture in ^AX^Lung-on-chip (D25) was stained for zonula occludens 1 (ZO-1; green). Nuclei were stained with DAPI (blue). Scale bar = 50 µm. **(D)** Representative staining of ^AX^iAECs in ALI condition on-chip show co-localization of HTII-280 and ABCA3 proteins on-chip. **(E)** Relative gene expression of distinct alveolar epithelial genes in ^AX^iAECs cultured in ALI culture compared to cells in submerged conditions (CTRL) on the AX12 (N = 1; *n* = 3). Data shown as mean ± SEM, *p < 0.05, **p < 0.01, ***p < 0.001.

A critical parameter for drug safety and toxicity studies *in vitro* is the recreation of a tight functional alveolar barrier (Zhang et al., 2018; Sapoznikov et al., 2019). To assess the biological impact of the alveolar microenvironment and investigate barrier integrity over time, ^AX^iAECs were cultured on the AX12 up to 25 days in submerged conditions. We set the threshold for a robust alveolar barrier at 1,000 Ω cm^2^, as this value is indicative of a tight monolayer for primary human alveolar cells with active water and ion transport (Fuchs et al., 2003). Our results demonstrated a tight barrier formation that reached the threshold at day 14–16 from cell seeding with a maximum reaching 3,000 ± 500 Ω cm^2^ (Figure 4A). Furthermore, cells exhibited a robust barrier function (TER) across passages initiating within a similar timeframe (Supplementary Figure S2D). ^AX^iAECs were additionally cultured in ALI conditions on the AX12 to replicate more faithfully the physiologically relevant alveolar barrier. Here, a strong barrier formation was again observed over time in the same time window as for submerged conditions, reaching maximal TER values of 2,300 ± 800 Ω cm^2^ (Figure 4B).

The alveolar barrier function of the lung epithelium depends on the orchestrated interactions among tight junctions, adherens junctions and the actin cytoskeleton (Fanning et al., 1998; Ivanov et al., 2010). Therefore, we further analyzed barrier formation on-chip by investigating the expression and distribution of tight junction protein ZO-1 by immunofluorescent staining in submerged and ALI conditions for 25 days. F-actin fibres were also examined by phalloidin staining (Supplementary Figure S2A). Our results demonstrated a tight ZO-1 network encompassing the ^AX^iAEC borders in both submerged and ALI conditions on-chip (Figure 4C). Interestingly, HTII-280 was detected co-localizing with ABCA3 in ^AX^iAECs cultured in ALI, indicative of AT2-like phenotype (Figure 4D and Supplementary Figure S3B). The gene expression analysis in ^AX^iAECs harvested from D25 on-chip confirmed a significant increase in transcript levels of AT1 cell-associated aquaporin 5 (AQP5) and AT2 cell makers (ABCA3, SFTPC) in ALI conditions compared to submerged cell culture conditions (Figure 4E). These results highlight the plasticity of the ^AX^iAECs-on-chip, which adopt an *in vivo* like phenotype when cultured in physiologically relevant conditions (ALI).

### Effect of Physiological Cyclic Stretch on Gene Regulation

**FIGURE 5:**
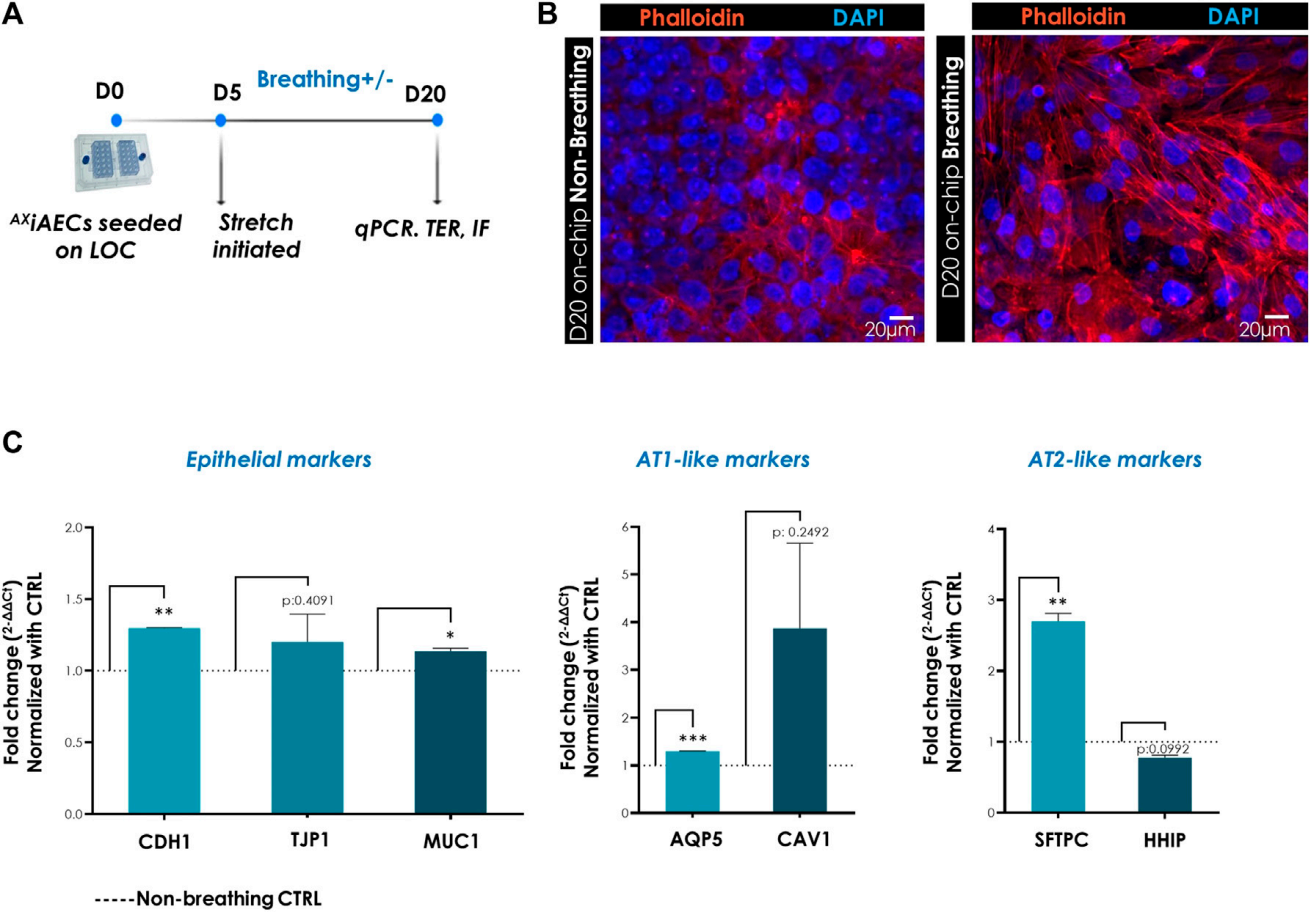
Breathing-induced differential gene expression in ^AX^iAECS on-chip. **(A)** Timeline and schematic of the ^AX^iAECs cultured in the ^AX^Lung-on-chip. Breathing was started at D5 on-chip. **(B)** Immunofluorescent stainings of ^AX^iAECs in breathing and non-breathing (CTRL) conditions, fixed after 20 days culture on AX12 and stained with phalloidin to visualize F-actin fibers (red). Nuclei were stained with DAPI (blue). Scale bar = 20 µm. **(C)** Normalized gene expression of epithelial cell associated, alveolar type I cell specific and alveolar type II cell specific markers following 20 days of culture in AX12. Data shown as mean ± SEM (N = 2; *n* = 4), *p < 0.05, **p < 0.01, ***p < 0.001.

To investigate the impact of *in vivo* like breathing, ^AX^iAECs on-chip were exposed to breathing (3D cyclic stretch, 10% linear strain, 0.2 Hz frequency) from D5 until D20 in submerged conditions (Figure 5A). One chip was cultured in breathing conditions starting on day 5 and the second chip of the AX12 plate was left in non-breathing control (CTRL) conditions (Figure 5A) for each experiment. Fifteen days post-stretch, cells were harvested and relative gene expression levels were compared between breathing and non-breathing conditions. To assess the involvement and reorganization of actin cytoskeletal filaments in response to biomechanical stretch, phalloidin staining was performed on the cells. In breathing conditions, cells displayed enhanced expression of F-actin filaments relative to cells in static culture conditions (Figure 5B). To obtain more detailed insight into the molecular alterations triggered by breathing conditions, differential gene expression was analyzed. Among the epithelial markers, the genes CDH1 and MUC1 (*p*-value 0.0201) were significantly overexpressed in breathing condition, whereas ZO-1 (*p*-value 0.4091) levels remained same (Figure 5C). The AT1 cell associated genes AQP5 (*p*-value 0.0002) and CAV1 (*p*-value 0.2492) exhibited an increasing trend in breathing cells (Figure 5C). The AT2 cell-specific gene coding for SFTPC (*p*-value 0.0043) also demonstrated a significant upregulation in breathing conditions relative to CTRL non-breathing cells, while HHIP (Hedgehog-Interacting Protein; *p*-value 0.992) remained unaltered (Figure 5C).

### TGFβ1 Pro-Fibrotic Induction in ^AX^iAECs On-Chip

**FIGURE 6:**
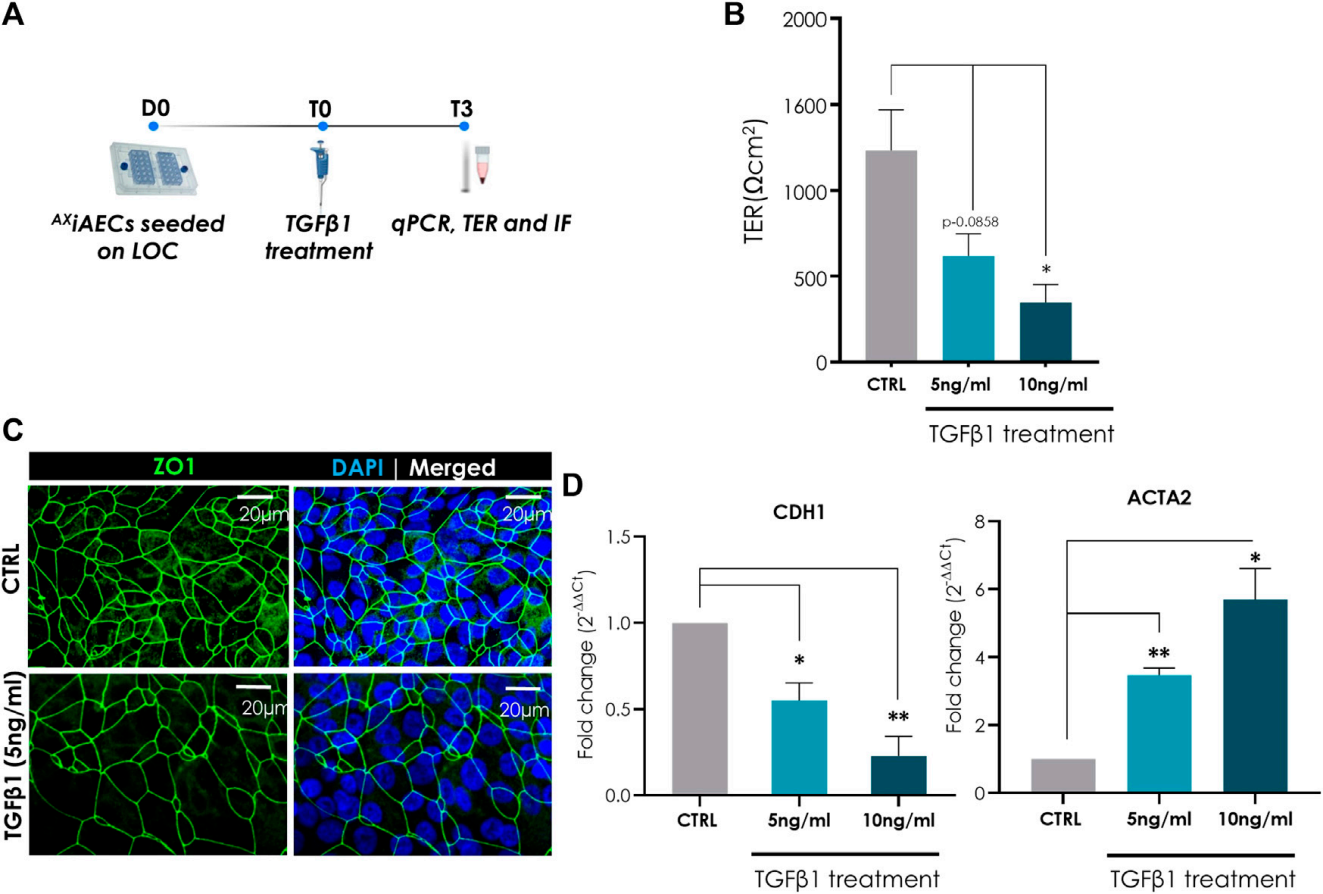
TGFβ1 treatment induced EMT in ^AX^iAECs on-chip. **(A)** Timeline and schematic of TGFβ1 treatment on day T0 in ^AX^iAECs. Cells were harvested and fixed for imaging 72 h (T3). **(B)** Significant dose-dependent reduction of TER (Ω cm^2^) values recorded on day T3 after treatment with TGFβ1. **(C)** Representative immunofluorescence staining of Control (untreated) and 5 ng/ml TGFβ1 treated cells (N = 2; *n* = 6). Cells fixed on day T3 and stained for ZO-1 (green). Reduced fluorescence intensity of ZO-1 observed in TGFβ1 (5 ng/ml) treated cells on-chip. **(D)** Normalized gene expression of EMT-associated cellular markers assessed following 3 days of treatment with TGFβ1 (N = 2; *n* = 4) on day T3. Data shown as mean ± SEM, *p < 0.05, **p < 0.01, ***p < 0.001.

To evaluate the application of the ^AX^iAECs on-chip model for lung injury, the pro-fibrotic mediator TGFβ1 was used to induce a fibrogenic response as it would occur during wound healing. For this, ^AX^iAECs were cultured on-chip until barrier formation and subsequently treated with 5 or 10 ng/ml TGFβ1 on day T0 for 72 h (T3) (Figure 6A). Absolute TER (Ω cm^2^) measured on day T3 demonstrate significant and dose-dependent decrease in TGFβ1 instilled cells, indicative of a disrupted barrier (Figure 6B). Furthermore, significant barrier disruption occurred from 24 h onward (Supplementary Figure S5B) for both concentrations of TGFβ1 used. Normalized fluorescence intensity assessment confirmed a trend in the reduction of ZO-1 expression in TGFβ1 treated cells compared to CTRL vector treated cells (Figure 6C; Supplementary Figure S5D). It is known that TGFβ1 treatment in AECs promotes epithelial-mesenchymal transition (EMT) *in vitro* (Kasai et al., 2005). To investigate this further, differential gene expression analysis was performed with TGFβ1 treated (5, 10 ng/ml) and CTRL cells harvested at day T3. Significant and concentration-dependent reduction of CDH1 gene expression (Rel. fold change for 5 ng/ml = 0.55; for 10 ng/ml = 0.23) (Figure 6D) and heightened levels of ACTA2 (α smooth muscle actin; Rel. fold change for 5 ng/ml = 3.47; for 10 ng/ml = 5.69) (Figure 6D) and COL1A1 (collagen 1α1; Rel. fold change for 5 ng/ml = 4.60; for 10 ng/ml = 4.69) (Supplementary Figure S5C) transcript levels were observed in TGFβ1 treated ^AX^iAECs on-chip, characteristic of an ongoing EMT mechanism.

### Influence of LPS Treatment in ^AX^iAECs On-Chip

Secondly, we evaluated the suitability of the ^AX^iAECs on-chip model in context of inflammation. We increased the model complexity to bring the system closer to the *in vivo* situation, where not only inflammation but also vascular leakage and edema were considered. To this end, ^AX^iAECs were co-cultured with endothelial cells (hLMVEC) and exposed to a commonly used proinflammatory trigger, bacterial LPS, in the presence of immune cells.

**FIGURE 7:**
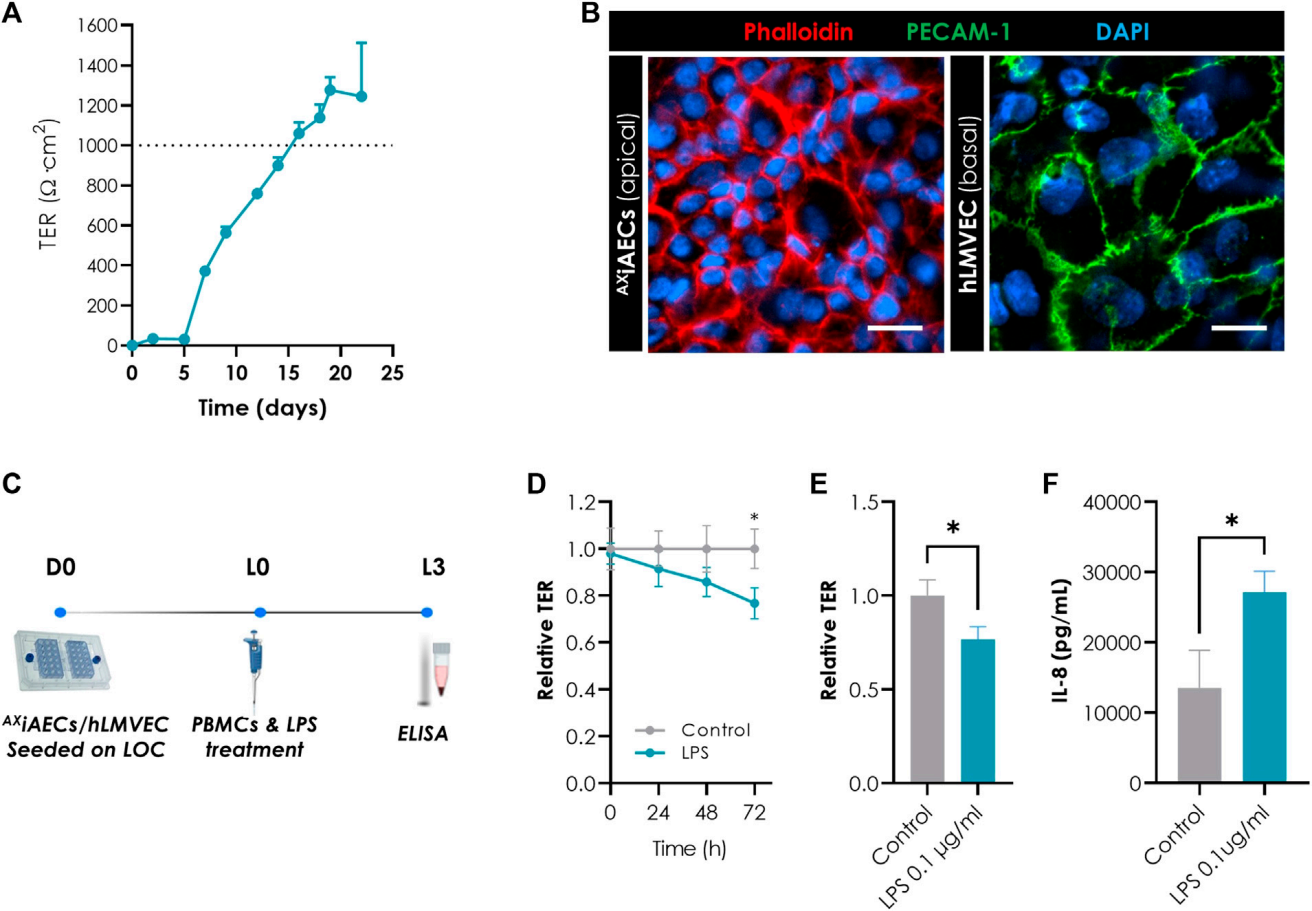
LPS-induced inflammation in ^AX^iAECs/hLMVEC co-culture on-chip. **(A)** TER values above 1,000 Ω cm^2^ (dotted line) were recorded from day 16 (N = 4, *n* = 42). **(B)** Representative image of ^AX^iAECs/hLMVEC co-culture stained with phalloidin (red) and PECAM-1 (green). Nuclei were stained with DAPI (blue). Scale bar is 20 µm. **(C)** Timeline and schematic of LPS treatment on day L0 in ^AX^iAECs/hLMVECs/PBMCs. Cells were harvested 72 h after stimulation with 0.1 μg/ml LPS (L3). **(D)** Significant reduction on TER values (relative to L0 and control conditions) recorded on day L3 after treatment with LPS (control, N = 3; *n* = 7; LPS treated: N = 3; *n* = 9). **(E)** IL-8 secretion from control (untreated) and LPS (0.1 μg/ml) treated cells were determined by ELISA on L3 (control, N = 3; *n* = 6; LPS, N = 3; *n* = 7). **(F)** IL-8 secretion from control (untreated) and LPS (0.1 μg/ml) treated cells were determined by ELISA on L3 (control,N = 3; *n* = 6; LPS, N = 3; *n* = 7). Data shown as mean ± SEM, *p < 0.05, **p < 0.01, ***p < 0.001.

The co-culture-on-chip model demonstrated a strong barrier formation with a rapid and sustained TER increase reaching more the barrier integrity threshold at D16 (1,000 Ω cm^2^) (Figure 7A). This model was characterized by a compact and homogenous epithelial cell layer with defined cell borders (Figure 7B), and endothelial cells displaying a typical cobblestone morphology decorated with PECAM-1 (CD31) at the cell membrane (Gaugler et al., 2004) (Figure 7B).

To simulate inflammation, the model was challenged with 0.1 μg/ml LPS after barrier formation (Figure 7C). At the time of treatment, PBMCs were introduced into the basolateral compartment to simulate cell-cell crosstalk in a proinflammatory setting. The treatment with LPS resulted in a time dependent drop in barrier function up to 72 h (L3) (Figures 7D,E). Upon barrier dysfunction, apical supernatants were collected and analyzed by ELISA to evaluate the impact at the epithelial compartment. Our results indicated a significant increase in the secretion of IL-8 cytokine levels for LPS stimulated cells relative to the control condition, pointing out the responsiveness of this co-culture model on-chip to proinflammatory stimuli (Figure 7F).

## Discussion

Alveolar barrier disruption is a hallmark event in fatal pulmonary conditions like ARDS, emphysema and idiopathic pulmonary fibrosis (Yanagi et al., 2015; Bärnthaler et al., 2017; Hou et al., 2019). The discovery of appropriate pharmaceutical compounds for such chronic diseases requires robust pre-clinical *in vitro* models that can mimic a disrupted alveolar barrier. Primary AECs remain the gold standard in terms of physiological relevance for modelling distal lung. However, they present some limitations, including donor to donor variability, scarcity, spontaneous differentiation *in vitro* and controversial use of “healthy” human tissue obtained from tumor resections. For this reason, continuous cell lines represent an interesting and suitable alternative for high throughput screening of toxic molecules or drugs in a preclinical context. Therefore, to ensure reproducibility, robustness, and stability of culture conditions, we characterized a novel distal airway model consisting of a new immortalized human alveolar epithelial cell line (^AX^iAECs) cultured in an *in vivo* like alveolar microenvironment (^AX^Lung-on-chip system).

^AX^iAECs cultured on-chip developed stable alveolar barrier function preserved across passages with an increased range of TER values (2,000–3,500 Ω cm^2^) in both mono and co-culture with endothelial cells (Figures 4, 7; Supplementary Figure S2D). Conversely, barrier forming features are not common for other established alveolar cell lines representative for AECs (van den Bogaard et al., 2009; Nalayanda et al., 2009). The formation of a stable alveolar barrier requires the combined cooperation of various junctional complexes especially tight junctions (like occludins, zonula occludens, claudins) adherens junctions (like cadherins) and actin cytoskeletal filaments (Ivanov et al., 2010; Campbell et al., 2017). Here, both ^AX^iAECs and A549 cells cultured on-chip (Day 7) demonstrated an increased expression of E-Cadherin (CDH1, Figure 3C) and ZO-1 (Figure 3C) compared to their levels at the time of seeding (Supplementary Figure S1C). Functionally however, ^AX^iAECs formed a tight barrier characterized by high TER values in contrast to A549, which do not reach barrier formation (Ren et al., 2016; Leibrock et al., 2019). Besides, A549 cells exhibited multilayer formation already from D7 (Supplementary Figure S2B), whereas, according to our findings, ^AX^iAECs retained a more stable structure even at later timepoints (Supplementary Figure S2C). Hence, despite both cellular models are promising considering the alveolar markers described here, the carcinoma origin of A549 cells, their inability of forming a TER-tight barrier and their quick multilayer formation, points out ^AX^iAECs on-chip as a more suitable model for the alveolar epithelial barrier. In terms of alveolar phenotype, several cell lines preserve some of the relevant alveolar epithelial markers at gene and protein levels. The recently established hAELVi cell line exhibits an ATI-like phenotype including high TER and tight junction formation, as well as caveolin-1 expression (Kuehn et al., 2016; Metz et al., 2020). However, hAELVi cells do not exhibit AT2 markers (such as SP-C) (Kuehn et al., 2016). AT2-associated pulmonary surfactant proteins (SP) and lipids play a key role in balancing respiratory dynamics and regulating alveolar inflammation (Cañadas et al., 2020), for instance by inhibiting the JAK/STAT activation pathway (Jin et al., 2018), binding LPS (SP-C) (Augusto et al., 2003) or opsonizing pathogens (SP-A, SP-D) (Crouch and Wright, 2001; Nayak et al., 2012). In this work, the AT1 cell specific HTI-56 and Homeobox only protein x (HOPX), a protein involved in adult lung alveolar injury and fibrosis progression (Ota et al., 2018), were observed on-chip at the protein level (Figure 3B). Other AT1 associated genes (AQP5, and CAV1, Figures 5C, 3C), were detected, consistent with the ^AX^iAECs-on-chip exhibiting an AT1-like phenotype. On the other hand, quantification of gene expression and immunofluorescence staining confirmed the long-term expression of SP-C, and proteins involved in surfactant metabolism (ABCA3) in the ^AX^iAECs cultured on the AX12 (Figures 3B–D). Although, expression of SFTPC in ^AX^iAECs was low at D0 (Supplementary Figure S1D) compared to whole lung and primary AT2 cells (Supplementary Figure S1B), a progressive and robust increase was observed over time on-chip at the protein and gene level (Figures 3B,D). Besides, breathing dynamics and ALI were also found to further enhance the expression of this AT2 marker (Figures 4E, 5C).

The impact of the lung-on-chip micro-environment as a driver for physiological lung markers was also observed for A549. Our results showed that these cells cultured on the ^AX^lung-on-chip displayed a significant increase of epithelial (CDH1) and alveolar (CAV1, ABCA3) markers (Supplementary Figures S1E,F) compared to culture on inserts within a similar time-frame. Altogether, our findings emphasize the crucial role of integrating physiological parameters in culture conditions.

We also investigated the presence of Mucin1 (Muc1), a membrane associated mucin released mainly by AT2 cells, with a minor presence in AT1 cells (Astarita et al., 2012). Recent studies have established that MUC1 or its released fragment, KL6 are prominent membrane markers mediating the expression of anti-inflammatory genes in interstitial lung diseases, asthma, COPD and lung cancer (Ishikawa et al., 2011; Ishikawa et al., 2012; Milara et al., 2018; Milara et al., 2019). Our results showed that ^AX^iAECs on-chip model expressed this clinically relevant target, at both protein and gene transcription levels (Figures 2, 3B,C), at higher levels than A549 cells on-chip (Figure 3C) indicating its potential applications to model lung disease.

Physiological cues including breathing and inter facial stresses are particularly relevant in the mature and developing lung (Waters et al., 2012; Knudsen and Ochs, 2018), and are crucial to recapitulate *in vivo* like functions in AECs (Singer et al., 2003; Stucki et al., 2018; Van Riet et al., 2020; Diem et al., 2020). In our model, we have demonstrated that physiological breathing conditions (3D stretch, 10% linear strain, 0.2 Hz) and ALI enhanced both barrier properties and the alveolar character of ^AX^iAECs-on-chip. Breathing-like stretch resulted in an increased expression of the junction complex associated genes CDH1 and ZO-1 (Figure 5C), indicating robust barrier formation consistent with previous findings on stretched AECs (Stucki et al., 2018; Huang et al., 2021). In addition, AT1 cell specific gene CAV1 and AT2 cell specific SFTPC transcript levels were also increased under breathing conditions, suggesting that stretch supports alveolar features and barrier function (Sanchez-Esteban et al., 2001; Diem et al., 2020).

ALI, is known to be relevant for surfactant release and alveolar cell function (Ravasio et al., 2011; Hobi et al., 2012). Therefore, lung models including this physiological parameter represent more faithfully the *in vivo* situation to better understand alveolar function and chronic lung diseases (Pezzulo et al., 2011; Van Riet et al., 2020). To investigate the effect of ALI on our model, ^AX^iAECs were cultured under ALI conditions for 2-weeks on the AX12 (Figure 4B). ALI-cultured ^AX^iAECs demonstrated a tight functional barrier in long term culture conditions with TER above 1,000 Ω cm^2^ (Figure 4B) consistent with previous observations on human AECs (Stucki et al., 2018; Huang et al., 2021). Furthermore, we found a significant upregulation of AT1 and AT2 markers (AQP5, SFTPC and ABCA3) in ALI culture compared to submerged conditions (Figure 4E), consistent with the preservation of alveolar markers of primary alveolar cells on ALI chips (Van Riet et al., 2020) and similar to the observations from a recent study on A549 (Wu et al., 2018). In this work, ALI conditions enhanced the alveolar character of this cell line, leading to an increased expression of AT1 and AT2 markers (Wu et al., 2018).

Research efforts over the past two decades have focused on streamlining drug development pipeline to reduce animal experimentations according to the 3R principles (replace, reduce and refine) (Ehrhardt and Kim, 2007). NAMs have gained a central role during the recent respiratory disease pandemic (COVID-19), accentuated by the limited recapitulation of critical features of COVID-19 pathogenesis in animal models (Kiener et al., 2021). Besides, it has been shown that life threatening forms of the disease (pneumonia, ARDS) result from the infection of AT2 cells in the distal lung (Mulay et al., 2020). These facts have added to the increasing need for predictive *in vitro* human models of the distal lung, for which conventional models, such as A549, have shown limited suitability in terms of infectivity or viral propagation (Si et al., 2021). On-chip approaches have proven to be efficient when recapitulating relevant COVID-19 hallmarks in the upper (Si et al., 2021) and in the distal lung: alveolar-capillary barrier, differential alveolar epithelial and endothelial host cell responses, replication of viral particles in the alveolar epithelium and increased inflammatory cytokine release (Zhang et al., 2020; Thacker et al., 2021). These studies have also provided insights into the molecular mechanisms of viral infection and replication relevant for drug repurposing (Si et al., 2021), as well as different cell targets in addition to AT2 cells, including HTII-280- ACE2+ cells (Zhang et al., 2020), or lung microvascular endothelial cells (Thacker et al., 2021). Here, we have demonstrated that our cell line on-chip model expresses key host cell factors for viral infection (ACE2, TMPRSS2, Furin and NRP1) (Hoffmann et al., 2020a; Hoffmann et al., 2020b; Kyrou et al., 2021) after 7 days of culture (Figure 3E), underlining the potential for the ^AX^iAECs on-chip model as a tool to investigate key pathways for productive infection with SARS-CoV-2.

To confirm the application of ^AX^iAECs on-chip as an *in vitro* model for disease modelling and toxicity screening, we exposed them to TGFβ1 or LPS to simulate key events during lung regeneration and inflammation. After TGFβ1 treatment, the alveolar barrier was significantly disrupted as measured by TER (Figure 6B; Supplementary Figure S5B) in agreement with previous studies (Pittet et al., 2001; Overgaard et al., 2015). TGFβ1 treatment is associated with the induction of Epithelial-mesenchymal transition (EMT), an intricate and orchestrated mechanism where epithelial cells lose their specific markers and adopt a mesenchymal cell phenotype in response to stress or injury (Kalluri and Weinberg, 2009). Until now, the occurrence of EMT in microfluidic models has only been reported in the context of cancer studies (Dhawan et al., 2018; Liu et al., 2020; Cho et al., 2021). Interestingly, our results suggest EMT occurrence in the ^AX^iAECs when treated with TGFβ1, which is evidenced by the decrease in the epithelial specific marker, CDH1, and the increase in mesenchymal cell targets such as ACTA2 and COL1A1 (Figure 6D; Supplementary Figure S5C). In addition, TGFβ1 treatment was found to induce the loss of ZO-1 in human epithelial cells (Zhang et al., 2013). Consistent with this work, reduced protein expression was observed in cells treated with TGFβ1 on-chip (Figure 6C). Future studies addressing the signaling pathway mechanism for TGFβ1 response at the alveolar epithelium would benefit from the inclusion of lung fibroblasts, a key player in profibrotic remodelling. These cells can be introduced in co-culture with ^AX^iAECs on-chip and used as a preclinical model for safety and efficacy testing as previously reported (de Maddalena et al., 2021).

Finally, the response of this cellular model to inflammatory stimuli was investigated. LPS was used as a pro-inflammatory stimulus in an advanced model of the air-blood barrier including ^AX^iAECs co-cultured with endothelial cells (hLMVECs). LPS treatment in the presence of the immune component (PBMCs), led to a significant disruption of the alveolar barrier after 72 h, which was observed by a decrease in TER as previously described (Roldan et al., 2019). This is consistent with a leakier air-blood barrier as observed in patients with sepsis-associated acute lung injury (Meng et al., 2010; Kobayashi et al., 2016). This decrease in TER was accompanied by an increase in proinflammatory cytokine IL-8 secretion from the alveolar epithelial cell compartment, as previously reported (Figure 7F) (Standiford et al., 1990; Witherden et al., 2004; Roldan et al., 2019), reflecting the crosstalk between the different players involved including the immune component and endothelial cells. IL-8 is a neutrophil chemoattractant chemokine (Henkels et al., 2010), suggesting that our *in vitro* observations may precede immune cell recruitment and neutrophil infiltration *in vivo*, which is associated with further barrier damage and inflammation and is a hallmark of various inflammatory lung diseases (Grommes and Soehnlein, 2010; Yang et al., 2021).

Altogether, our results point out that physiologically mimicking cell culture conditions are essential to recapitulate crucial features of alveolar AT1 and AT2 cells *in vitro*. More specifically, we have shown that long term culture, breathing and ALI conditions provided by the chip technology support an enhancement of AT1- and AT2-like phenotypes on the ^AX^iAECs cultured on-chip, resembling functional coexistence. Recent studies highlight the relevance of simulating the *in vivo* lung microenvironment (structure, cell types and mechanical cues) (Ainslie et al., 2019; Nawroth et al., 2019) and the key role of mechanical forces during lung development and function (Waters et al., 2012). Along with those works, our data reinforces the essential role of easy-to-use organ-on-chip technology combined with well-characterized and physiological functioning proper cell types to tackle the need for predictive preclinical human models. These models are beneficial for basic research, but additionally, have precise importance for industrial applications, including toxicity testing of inhaled molecules, and drug safety and efficacy studies. Particularly in the industrial setting, cell line-based organs-on-chip such as the ^AX^iAECs on-^AX^Lung-on-chip represent a path forward to provide qualified models with higher throughput, robustness, and automatization opportunities, which are essential factors for the standardization and validation of this technology with a regulatory scope (Ainslie et al., 2019; Clapp et al., 2021; Rusyn and Roth, 2021).

The future addition of other cellular components like alveolar macrophages (Mubarak et al., 2018), or modelling inhalation exposure *in vitro*, will be of high relevance to further establish this technology as a reference NAM for xenobiotics toxicity assessments, as well as for drug safety and efficacy applications (Artzy-Schnirman et al., 2019; Ding et al., 2020; Sengupta et al., 2021).

## Conclusion

In summary, the breathing ^AX^iAECs on-chip model recapitulates critical aspects of the alveolar microenvironment including air-blood barrier function, the breathing motion and long-term culture conditions in ALI for cell differentiation. The new cell line ^AX^iAECs on-chip, forms a tight barrier with TER values above 1,000 Ω cm^2^, expresses key AT1 and AT2 markers and SARS-CoV-2 infection-associated cellular host factors. Besides, we have demonstrated that this model responds to profibrotic and proinflammatory triggers that elicit physiological responses such as EMT and inflammation, respectively.

In addition to simple handling, this model allows reproducible cell culture conditions that are pivotal for drug-development and toxicity screening studies. Altogether, our current results suggest that this alveolar barrier on-chip model will be a valuable tool for precision medicine applications in the future and is a promising alternative to animal models currently used for respiratory research.

## Data Availability

The original contributions presented in the study are included in the article/Supplementary Material, further inquiries can be directed to the corresponding author.

## Supplementary Material

Supplementary Figure S1 Differential gene expression of ^AX^iAECs and A549 cells. **(A)** Relative epithelial and **(B)** alveolar gene expression of freshly isolated primary AECs (N = 2 donors; *n* = 4) and from resected healthy human lung tissues (Whole lung; N = 1; *n* = 2). **(C)** Comparative qPCR reveals distinct epithelial and **(D)** alveolar gene expression levels in A549 cells (N = 1; *n* = 3) and ^AX^iAECs at Day 0 (N = 2; *n* = 4). A549 cells cultured on AX12 for 7 days (N = 1; *n* = 3) show improved **(E)** epithelial and **(F)** alveolar marker levels compared to when cultured on inserts for 7 days (N = 1; *n* = 3). Data shown as mean ± SEM, **p* < 0.05, ***p* < 0.01, ****p* < 0.001.

Supplementary Figure S2 Characterization of A549 cells on ^AX^lung-on-chip. **(A)** Representative immunofluorescent stainings of Epcam (yellow), HT1-56 (green), ABCA3 (red) and DAPI (blue) in A549 cells, fixed after 7 days cultured on ^AX^Lung-on-chip. **(B)** Orthogonal view for A549 cells on-chip (HT1-56 in green, DAPI in blue) at Day 7, **(C)** Orthogonal view for ^AX^iAECs (actin in orange, DAPI in blue) at Day 14 on-chip. Scale bar = 20 µm. **(D)** TER progression was measured across increasing passages (P27–P31) of ^AX^iAECs.

Supplementary Figure S3 Air-liquid interface culture conditions on-chip improve the alveolar character of ^AX^iAECs. **(A)** Representative immunofluorescent images for ^AX^iAECs on-chip stained for phalloidin (red) in ALI conditions show a tight network of actin filaments, fixed after 25 days culture (D25). Nuclei were stained with DAPI (blue). **(B)** Immunofluorescent stainings of ^AX^iAECs in ALI condition on-chip demonstrate co-localization of HTII-280 (red) and ABCA3 (green) on-chip. Scale bars provided with each image.

Supplementary Figure S4 Breathing induced gene expression changes of SARS-CoV-2 host factors in ^AX^iAECs on-chip. **(A)** Timeline and schematic of the ^AX^iAECs cultured in the ^AX^Lung-on-chip. Breathing was started at D5 on-chip. **(B)** Normalized qPCR data show gene expression of SARS-CoV-2 associated markers following 20 days of culture in AX12. Data shown as mean ± SEM (N = 1; *n* = 2).

Supplementary Figure S5 Time-course investigation of alveolar barrier changes in TGFβ1 treated ^AX^iAECS on-chip. **(A)** Timeline and schematic of TGFβ1 treatment on day T0 in ^AX^iAECs. TER (Ω cm^2^) was measured every 24 h until day T3 after treatment. **(B)** Reduced TER (Ω cm^2^) values, normalized to day T0 (before TGFβ1 treatment) were recorded until day T3 in cells treated with 5 and 10 ng/ml TGFβ1 (N = 1; *n* = 3). **(C)** Relative gene expression of collagen 1α1 (COL1A1) was assessed in cells following 3 days of treatment with TGFβ1 (N = 1; *n* = 3). Data shown as mean ± SEM, **p* < 0.05, ***p* < 0.01, ****p* < 0.001.

Supplementary Table S6 Primer sequences.
