# Supplementary figures and images for: A New Immortalized Human Alveolar Epithelial Cell Model to Study Lung Injury and Toxicity on a Breathing Lung-On-Chip System
Source: Front Toxicol. 2022 Jun 17;4:840606. doi: 10.3389/ftox.2022.840606 (PMC9272139; doi:10.3389/ftox.2022.840606)

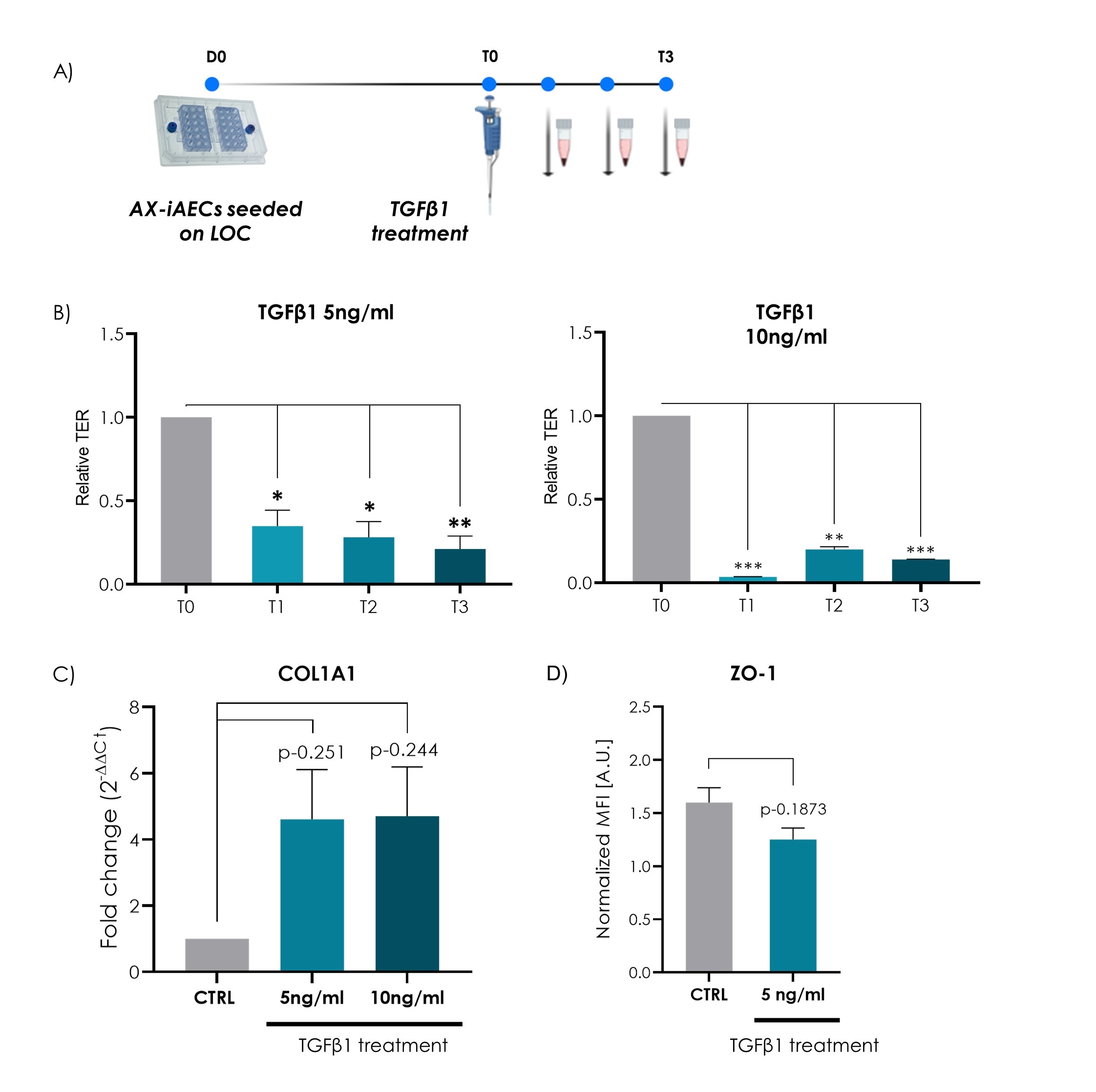

Supplement: Supplementary file 1 [file Image5.jpg]

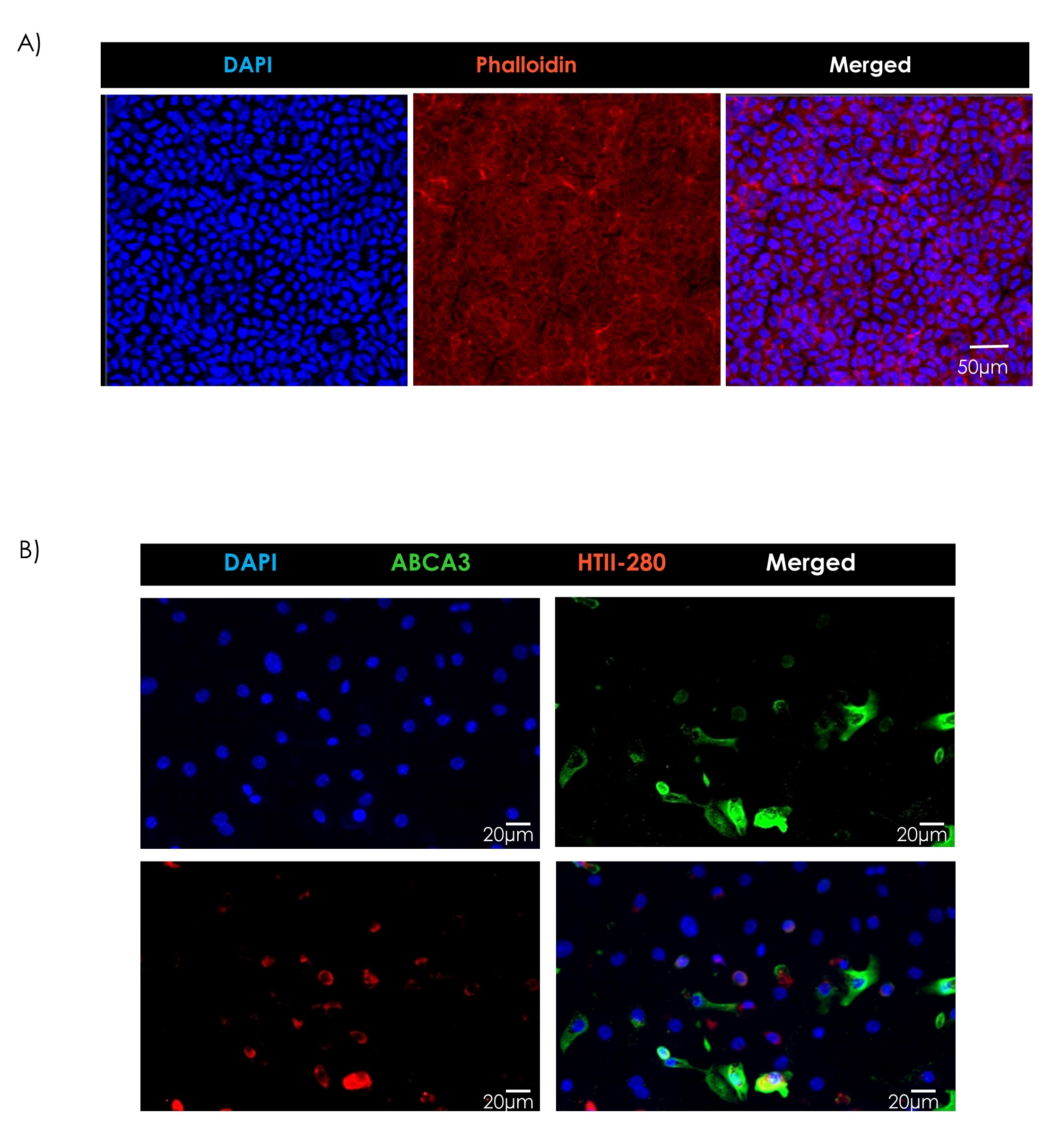

Supplement: Supplementary file 2 [file Image3.jpg]

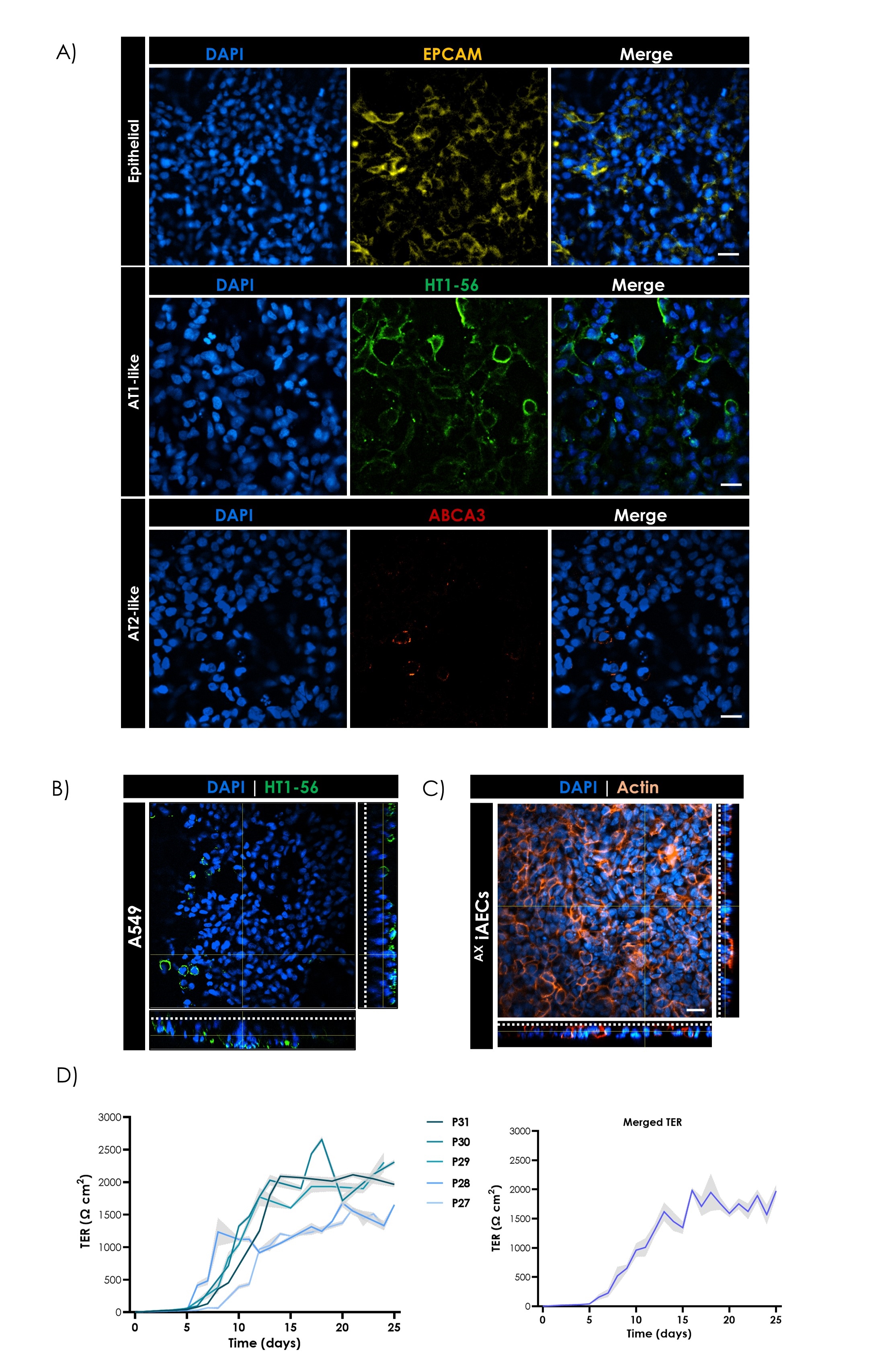

Supplement: Supplementary file 3 [file Image2.jpg]

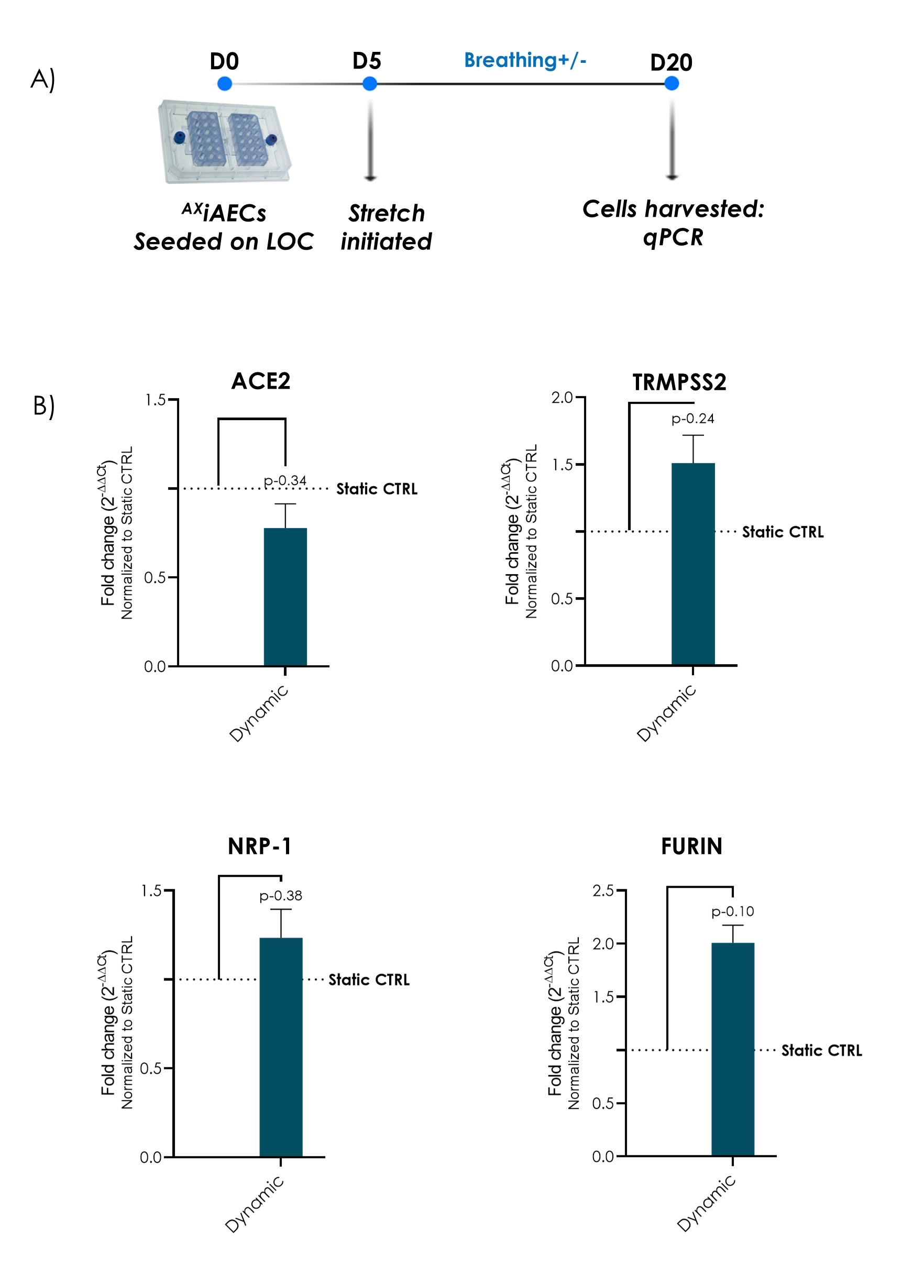

Supplement: Supplementary file 5 [file Image4.jpg]

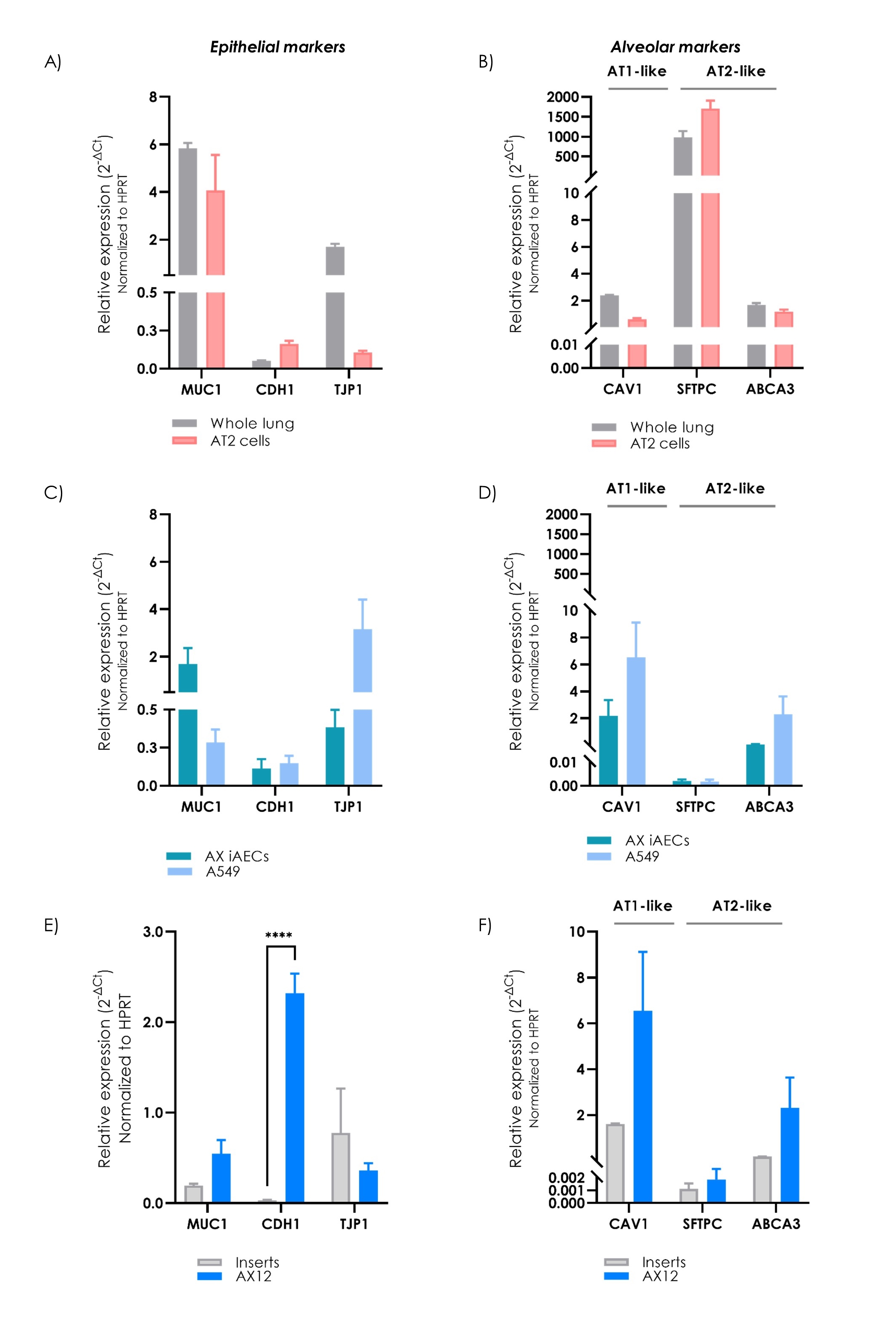

Supplement: Supplementary file 6 [file Image1.jpg]
