# Supplementary material for: A New Immortalized Human Alveolar Epithelial Cell Model to Study Lung Injury and Toxicity on a Breathing Lung-On-Chip System
Source: Front Toxicol. 2022 Jun 17;4:840606. doi: 10.3389/ftox.2022.840606 (PMC9272139; doi:10.3389/ftox.2022.840606)
Supplement: Supplementary file 4 [file Table1.pdf]

Gene name	Forward primer (5'-3')	Reverse primer (5'-3')
CDH1	CGAGAGCTACACGTTCACGG	CGAGAGCTACACGTTCACGG
ZO1	CCCCACTCTGAAAATGAGGA	ACAGCAATGGAGGAAACAGC
MUC1	TCAGCTTCTACTCTGGTGCACAA	ATTGAGAATGGAGTGCTCTTGCT
SPC	ACAATCACCACGACGATGAG	AGCAAAGAGGTCCTGATGGA
ABCA3	CTCGCTGTTCCTCAAGCAGA	CTTCAAACCTGTGCGTGCTC
COL1A1	ATCAACCGGAGGAATTTCCGT	CACCAGGACGACCAGGTTTTC
AQP5	GCCACCTTGTCGGAATCTACT	GGCTCATACGTGCCTTTGATG
CAV1	AACGATGACGTGGTCAAGATTG	TCCAAATGCCGTCAAAACTGT
ACTA2	CAGGGCTGTTTTCCCATCCAT	GCCATGTTCTATCGGGTACTT
ACE2	CGAAGCCGAAGACCTGTTCTA	GGGCAAGTGTGGACTGTTCC
TRMPSS2	GGACAGTGTGCACCTCAAAGAC	TCCCACGAGGAAGGTCCC
FURIN	TCGGGGACTATTACCACTTCTG	CCAGCCACTGTACTTGAGGC
NRP1	CCCGGGTACCTTACATCTCCT	ACAGAACTTTCCCCTAAAATGTCC
HPRT	AGACTTTGCTTTCCTTGGTCAGG	GTCTGGCTTATATCCAACACTTCG
